# Intuitive physics learning in a deep-learning model inspired by developmental psychology

**DOI:** 10.1038/s41562-022-01394-8

**Published:** 2022-07-11

**Authors:** Luis S. Piloto, Ari Weinstein, Peter Battaglia, Matthew Botvinick

**Affiliations:** 1 DeepMind, London, UK; 2 Princeton Neuroscience Institute, Princeton University, Princeton, NJ USA; 3 Gatsby Computational Neuroscience Unit, University College London, London, UK

**Keywords:** Psychology, Cognitive neuroscience, Computational models, Philosophy

## Abstract

‘Intuitive physics’ enables our pragmatic engagement with the physical world and forms a key component of ‘common sense’ aspects of thought. Current artificial intelligence systems pale in their understanding of intuitive physics, in comparison to even very young children. Here we address this gap between humans and machines by drawing on the field of developmental psychology. First, we introduce and open-source a machine-learning dataset designed to evaluate conceptual understanding of intuitive physics, adopting the violation-of-expectation (VoE) paradigm from developmental psychology. Second, we build a deep-learning system that learns intuitive physics directly from visual data, inspired by studies of visual cognition in children. We demonstrate that our model can learn a diverse set of physical concepts, which depends critically on object-level representations, consistent with findings from developmental psychology. We consider the implications of these results both for AI and for research on human cognition.

## Abstract (web summary)

Piloto et al. introduce a deep-learning system which is able to learn basic rules of the physical world, such as object solidity and persistence.

## Main

The field of artificial intelligence (AI) has made astonishing progress in recent years, mastering an increasing range of tasks that now include Atari video games^1^, board games such as chess and Go^2^, scientific problems including protein-folding^3^ and language modelling^4^. At the same time, success in these narrow domains has made it increasingly clear that something fundamental is still missing. In particular, state-of-the-art AI systems still struggle to capture the ‘common sense’ knowledge that guides prediction, inference and action in everyday human scenarios^5,6^. In the present work, we focus on one particular domain of common-sense knowledge: intuitive physics, the network of concepts that underlies reasoning about the properties and interactions of macroscopic objects^7^. Intuitive physics is fundamental to embodied intelligence, most obviously because it is essential to all practical action, but also because it provides one foundation for conceptual knowledge and compositional representation in general^8^. Despite considerable effort, however, recent advances in AI have yet to yield a system that displays an understanding of intuitive physics comparable to that of even very young children.

To pursue richer common sense physical intuition in AI systems, we take, at multiple points in our work, inspiration from developmental psychology, where the acquisition of intuitive physics knowledge has been an intensive focus of study^9–12^. We build a deep-learning system that integrates a central insight of the developmental literature, which is that physics is understood at the level of discrete objects and their interactions. We also draw on developmental psychology in a second way, which relates to the problem of behaviourally probing whether an AI system (or in the case of developmental psychology, an infant or child) possesses knowledge of intuitive physics.

In developing behavioural probes for research on children, developmental psychologists have based their approach on two principles. First, that the core of intuitive physics rests upon a set of discrete concepts^11,13^ (for example, object permanence, object solidity, continuity and so on) that can be differentiated, operationalized and individually probed. By specifically targeting discrete concepts, our work is quite different from standard approaches in AI for learning intuitive physics, which measure progress via video or state prediction^14–16^ metrics, binary outcome prediction^17^, question-answering performance^18,19^ or high reward in reinforcement learning tasks^20^. These alternative approaches intuitively seem to require an understanding of some aspects of intuitive physics, but do not clearly operationalize or strategically probe an explicit set of such concepts.

The second principle used by developmental psychologists for probing physical concepts is that possession of a physical concept corresponds to forming a set of expectations about how the future can unfold. If human viewers have the concept of object permanence^21^, then they will expect that objects will not ‘wink out of existence’ when they are out of sight. If they expect that objects will not interpenetrate one another, then they have the concept of solidity^22^. If they expect that objects will not magically teleport from one place to another but instead trace continuous paths through time and space, then they have the concept of continuity^11^. With this conceptual scaffolding, a method for measuring knowledge of a specific physical concept emerges: the violation-of-expectation (VoE) paradigm^21^.

**Fig. 1:**
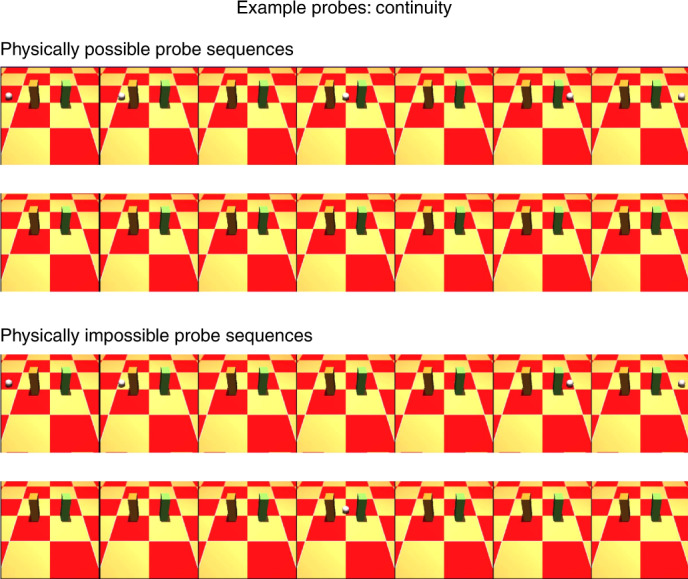
Probes adapted from developmental psychology to assess the physical concept of continuity. Example probes adapted from ref. ^26^ to assess the physical concept of continuity^11,69^: objects trace a continuous path through time and space. Each row corresponds to one temporally downsampled video in a probe tuple. Checkered backgrounds were used as a cue for depth and to introduce visual diversity of our stimuli. Actual videos consist of a total of 15 frames. The top two rows are physically possible probes and the bottom two rows are physically impossible probes. Physically possible probes: in the first physically possible probe (first row), a ball rolls behind two occluders. In the second possible probe (second row), no ball is present. Physically impossible probes: these probes are formed by splicing parts of the physically possible probes into impossible events. In the first impossible probe (third row), the ball rolls behind the first occluder and emerges from the second occluder, never appearing between the two occluders. The second impossible probe (fourth row) has the opposite structure: the ball appears between the two occluders, but was never seen rolling behind the first occluder or rolling out of the second occluder.

Using the VoE paradigm to probe for a specific concept, researchers show infants visually similar arrays (called probes) that are either consistent (physically possible) or inconsistent (physically impossible) with that physical concept. If infants are more surprised by the impossible array, this provides evidence that their expectations, derived from their knowledge of the probed physical concept, were violated. In this paradigm, surprise is putatively measured via gaze duration, but see refs. ^23–25^ for further discussion. As an example, consider the concept of continuity (depicted in Fig. 1): objects trace a continuous path through time and space. For the possible probe (Fig. 1, first row), researchers^26^ showed an object moving horizontally behind a pillar, being occluded by that pillar, subsequently emerging from occlusion, and travelling towards a second pillar, where it was again occluded behind that pillar and emerged from occlusion one final time. In the impossible probe (Fig. 1, third row), when the object is occluded by the first pillar, it does not emerge from occlusion immediately. Instead, after some delay, the object emerges from behind the second pillar - never appearing in the space between the two pillars and thus seeming to teleport from one pillar to the other. Experiments with infants have shown that by the age of 2.5 months, they gaze longer at an object that teleports between two screens than an object that moves continuously from one screen to the next^26^. This same strategy has been used by developmental researchers to accumulate strong evidence that infants acquire a wide range of distinct physical concepts^9–12^ within the first year of life.

In previous work^27^, we introduced a machine-learning video dataset designed to systematically test how well models can learn specific physical concepts (see Discussion for comparison against similar datasets developed in parallel^28^ and subsequently^29^). Our original dataset contained procedurally generated VoE probes that assess acquisition of a set of physical concepts, constructed so that no individual video frame could explain any resulting VoE effect. In the present work, we introduce a much richer video corpus, the Physical Concepts dataset (https://github.com/deepmind/physical_concepts). This new dataset contains VoE probe videos targeting five physical concepts that have been identified as central in the developmental psychology literature. The first three, continuity (Fig. 1), object persistence (Supplementary Fig. 1) and solidity (Supplementary Fig. 2), were introduced above. The fourth concept, ‘unchangeableness’ (refs. ^9,30^, Supplementary Fig. 3), captures the notion that certain object properties (for example, shape) do not change. The fifth and final concept, directional inertia (a more specific form of the inertia principle tested in ref. ^31^, Supplementary Fig. 4), involves the expectation that moving objects undergo changes in direction consistent with the principles of inertia. As detailed in Methods, each video showing a violation of these physical principles is matched with a corresponding video that provides a physics-consistent baseline, maintaining precisely the same single-image statistics across videos. Our probe videos were based on stimuli used in specific developmental psychology experiments, but we made changes to event details introduced to enhance experimental control without changing the span of physical concepts probed.

**Fig. 2:**
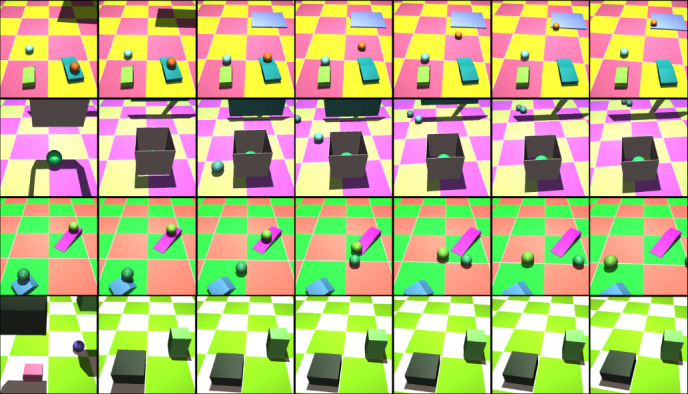
Videos from the ‘freeform’ data used to train our models. Example training videos (temporally downsampled, actual videos contain 15 frames). Scenes are constructed procedurally with composable interactions: objects added to a scene are either initialized completely randomly or target preexisting objects in the scene. The camera starts from a fixed location and drifts randomly over time.

Critically, the Physical Concepts dataset also includes a separate corpus of videos intended as training data. These videos show a wide variety of procedurally generated physical events (Fig. 2 and Methods) involving objects similar to those involved in the probe videos, among others, but never showing the specific events involved in the test probes. Readers are encouraged to view examples in video format: http://tiny.cc/phys_concepts_training.

**Fig. 3:**
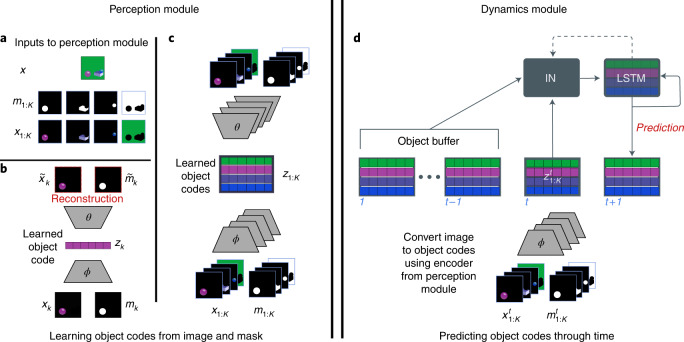
PLATO uses both a perceptual model and a dynamics model to make per-object predictions. PLATO consists of two components: the perception module (left) and the dynamics predictor (right). The perception module is used to convert visual input into a set of object codes. These object codes are used by the dynamics modules to predict future frames. **a**, The perception module takes as input an image *x* and an associated segmentation mask *m*_1:*K*_. Taking the elementwise product yields a set of images of just the visible parts of each object: *x*_1:*K*_. **b**, Given an object image-mask pair, the perception module produces an object code *z*_*k*_ via an encoder module *ϕ*. The object code is decoded back into a reconstruction of the object image-mask pair via the decoder module *θ*. The discrepancy between the reconstruction and the original image-mask pair is used to train the parameters of *ϕ* and *θ* such that *z*_*k*_ comes to represent informative aspects of each object image-mask pair. **c**, After training, an entire image can be reconstructed via a set of object codes *z*_1:*K*_ by independently running each image-mask pair through *ϕ* and decoding via *θ*. **d**, The dynamics module is trained on sequence data produced by running videos (and their segmentation masks) through the pretrained encoder *ϕ*. The dynamics module must predict the object codes in the next frame given the object codes in the current frame ${z}_{1:K}^{t}$ and an object buffer of the codes in the preceding frames ${z}_{1:K}^{1:t-1}$. The dynamics module comprises two trainable components: a ‘slotted’ object-based LSTM and an interaction network (IN). Predictions are made by computing interactions from each slot in the LSTM’s previous state (dotted arrow) to every other slot in the LSTM and all input object codes and buffers ${z}_{1:K}^{1:t}$. The resulting interaction is used to make objectwise predictions and updates to the LSTM.

Equipped with this dataset and evaluation framework, we now turn to the primary objective of the present research: to build a model capable of learning intuitive physics and dissect what enables that capacity. Our architecture is inspired by accounts from developmental psychology which posit that three object-centric processes underpin infant intuitive physics behaviour^9^. We leverage recent advances in AI to instantiate these systems in a model that we nickname PLATO, for Physics Learning through Auto-encoding and Tracking Objects. First and foremost is the process of object individuation^11^. Object individuation carves the continuous perceptual input of vision into a discrete set of entities, where each entity has a corresponding set of attributes. In PLATO, each segmented video frame is decomposed into a set of object codes via a perception module (Fig. 3a–c), thus implementing a mapping from visual input to individuated objects. PLATO does not learn to segment the scene (that task is accomplished via ground truth segmentation masks from the dataset), but given a segmented object learns a compressed representation. Second, object tracking (or object indexing) assigns an index to each object, enabling a correspondence between object percepts across time^32–34^ and computation of dynamic properties (Fig. 3b,c). In PLATO, the object codes are accumulated and tracked over frames in an object buffer (Fig. 3d). This is accomplished again by virtue of ground truth segmentation masks which provide a correspondence between objects across frames. The final component is relational processing of these tracked objects. This is inspired by the idea, proposed in developmental psychology, of a ‘physical reasoning system’^9^, which dynamically processes representations of objects, yielding new representations that are inflected by their relationship to and interactions with other objects. In PLATO, we learn interactions (Fig. 3d) between the object memory (a slotted object-based long short-term memory (LSTM)^35^) and the history of object percepts (the object buffer) to produce per-object predictions for the next video frame and update the object-based memory. We train PLATO on a next-step prediction task and evaluate its performance on our suite of intuitive physics probes. Although PLATO is unique in its detailed inspiration from the developmental literature and the domain to which it is applied, it is important to note that there are various similar models and proposals that prioritize object-centric representations, interactions and computations (for example, refs. ^20,36,37^).

To foreshadow our results, we find that PLATO displays strong VoE effects across all five concept-probe categories in our dataset. By contrast, carefully controlled comparison models that lack object-centred representations fail to achieve above-chance results on the evaluation suite, even when furnished with more computational capacity. Furthermore, we report that our object-centred model, when given object segmentation and tracking, can develop robust VoE effects with a surprisingly small amount of training data, equivalent to 28 h of visual experience. Finally, we evaluate our model’s behaviour on unseen objects and events as a strong test of generalization. We test PLATO, without additional training, on an independently developed test set, and find that it continues to display robust VoE effects in this generalization setting.

### Results

#### Models

To implement the object-centric approach, our model comprises two main components: a feedforward perceptual module (Fig. 3a–c) and a recurrent dynamics predictor with per-object memory (Fig. 3d). The perceptual module takes as input an image and segmentation mask—we discuss later how segmentations can also be learned from scratch from visual data—and converts these into a vector embedding using standard deep-learning auto-encoding methods (Fig. 3a–c and Methods). In effect, the perceptual module parses the high-dimensional visual input into a small set of discrete object codes.

The dynamics predictor centres on a structured recurrent neural network we developed called an InteractionLSTM (Fig. 3d: ‘IN’ and ‘LSTM’ boxes; Methods). This takes as input the history of single-frame object-level embeddings (Fig. 3, ‘object buffer’) and predicts the set of object codes at the next timestep (Fig. 3, ‘prediction’).

We hypothesized, on the basis of the insights provided by developmental psychology, that the involvement of object-level coding would be critical to the acquisition of intuitive physics concepts and corresponding VoE effects. To evaluate this claim, we constructed a well-matched object-agnostic ‘flat’ model as a baseline for comparison. For this, we took each component in PLATO that used a set of object codes, and replaced that set with a single vector embedding for the entire scene (Methods). We investigate two versions of the resulting flat model on the basis that we can match either the number of tunable parameters in PLATO or the number of units representing each video frame. In the flat equal parameters (FEP) baseline, the flat embedding has the same number of dimensions (16) as one of PLATO’s single-object codes. This model has the same number of learnable parameters in the dynamics predictor as PLATO, but reduced capacity for representing images in the perceptual module. In the second version, flat equal capacity (FEC), we give the flat code the same capacity for representing images in the perceptual model (a single 128-dimensional vector, given that PLATO contains eight 16-dimensional object-code slots). In this case the dynamics predictor contains approximately four million more parameters than PLATO (see Methods for full implementational details).

#### Training and testing

For all models, training consisted of two phases. In the first phase (Fig. 3a–c), the perceptual module was trained to reconstruct individual images from the training data (see Methods for loss function and other details). After this phase, the weights within the perceptual module were fixed, yielding an encoder that could produce object (or flat) codes from inputs and a decoder that could take object (or flat) codes and produce an image. In the second phase (Fig. 3d), we trained the dynamics module to predict the next set of object codes in videos from our training set (using teacher-forcing as detailed in Methods).

**Fig. 4:**
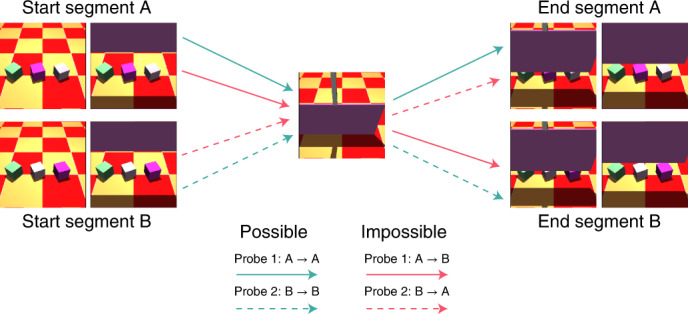
Illustration of the splicing procedure used to create our physical concept probes. Illustration of the splicing procedure used to create a probe tuple for the physical concept of ‘unchangeableness’ as seen in Supplementary Fig. 4. Probe tuples consist of two possible videos and two impossible videos. The possible videos are created by running a physics engine with related initial conditions (for example, same objects but different positions). Importantly, videos are constructed so that they share a frame in common (centre image). The segments before this shared frame are called the ‘start segments’ (left) and the ones after are called the ‘end segments’ (right). Thus, to create any probe, we can gather frames from a start segment, add the common frame and add frames from an end segment. Possible probes (green arrows) are formed by starting and ending at corresponding start and end segments (for example, start segment A to end segment A). Impossible probes (red arrows) are formed by using a mismatched end segment (for example, start segment A to end segment B). This design ensures that the possible videos and impossible videos within a probe tuple are perfectly matched in terms of individual frames and pairs of frames.

We evaluated our models using the VoE paradigm for the five physical concepts targeted in the Physical Concepts test set: continuity, directional inertia, object persistence, solidity and unchangeableness (see Methods for description). We chose these physical concepts to satisfy three criteria. First, we wanted to cover real-world physical phenomena (for example, gravity, solidity, stability and so on), which could be faithfully instantiated in a simulated environment. Second, we chose concepts that had some experimental precedence in the developmental literature. Third, we chose physical concepts that were amenable to the probe construction splicing procedure described below. For a single physical concept, we procedurally generated 5,000 probe tuples, each comprising two physically possible probes and two physically impossible probes. The impossible probes were constructed by splicing (illustrated in Fig. 4) together frames from the possible probes in a way that clearly violates physics. This splicing procedure ensures that the same set of images is present in the possible probes as in the impossible probes. The only difference is the ordering (which yields aphysical events in the impossible probes), an approach pioneered by Riochet et al.^28^. Furthermore, we splice frames such that all adjacent frames are physically possible, even if the scene as a whole is not. This ensures that the exact same pairs of images appear in both probe types. This precludes a model from showing strong VoE effects merely on the basis of temporally local inconsistencies (see Supplementary Fig. 17 for empirical validation).

Under the VoE paradigm, physical concept acquisition is quantified by comparing surprise on the two probe types. To calculate surprise for a given video, we compute for each frame the model’s prediction error, defined as the sum-squared error of the system’s pixel-level prediction. Then, we sum prediction errors across all frames within a video. For each of the 5,000 probe tuples for a physical concept, we compute the sum of the surprises on the possible probes, called the physically possible surprise, and similarly compute the physically impossible surprise. We compute an accuracy score where a probe is ‘classified’ correctly if the impossible surprise is greater than the possible surprise. We use the average accuracy to assess the model’s acquisition of a physical concept. Whereas accuracy is binary, we can also compute the relative surprise, the difference between the impossible surprise and possible surprise, to quantify the magnitude of the surprise effect. To allow for comparison across the probe tuples, we normalize the relative surprise by the sum of both the possible and impossible surprises. This normalization takes account of the fact that some initial conditions yield higher baseline surprises across both probe types (for example, probes with higher velocities). Finally, to accommodate variability in simulation results, we computed average accuracy and average relative surprise for five different initial random seeds of each model.

#### Test set performance

**Fig. 5:**
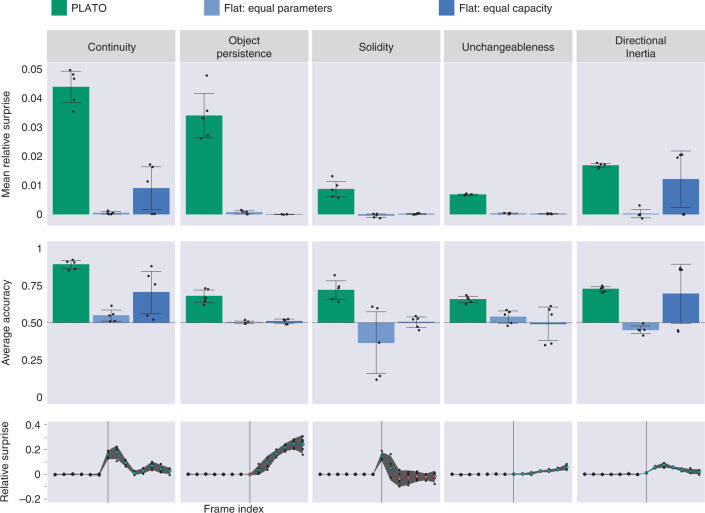
PLATO displays robust effects across the probes in our dataset. Top, middle rows: Comparison of object-based model PLATO (green) against flat baseline models (blue). PLATO consistently shows large effect sizes (top) in response to impossible events and robustly classifies them as impossible (middle). Even when the flat model has more model parameters (dark blue), it only shows above-chance accuracy on two of the five concepts. Each point represents the average performance over 5,000 probe tuples for models trained with different random seeds. Error bars show the 95% confidence intervals over five seeds, assuming a normal distribution across seeds (although not formally tested). Bottom**:** frame-by-frame analysis of surprise reveals that the relative surprise increases substantially when videos become physically impossible. The trajectory of this increase is specific to the physical phenomena. For example, in the ‘unchangeableness’ dataset the occluder rises slowly, which yields a gradual increase in relative surprise. In contrast, ‘continuity’ shows a steep peak at the frames where the ball fails to appear between the pillars. Relative surprise is computed at each frame of probe videos for five random seeds of the PLATO model. The *x* axis indicates frame index within a video. Vertical lines indicate when the impossible probes become aphysical (varies by dataset). Blue and red dots code points above or below zero, respectively, only applied to frames after the onset of aphysicality. Small dots depict different random seeds. Large dots depict mean over three seeds, with corresponding error bars showing 95% confidence intervals over seeds (assuming a normal distribution, but not formally tested).

At test time, PLATO displayed robust VoE effects in all five probe categories when trained with five different random seeds each evaluated over 5,000 probe quadruplets. This was evident both in the relative surprise effect, most analogous to the original developmental experiments (Fig. 5 top row, green bars) as well as in classification accuracy (Fig. 5 middle row, green bars).

We performed a one-tailed, single-sample *t*-test to assess whether the mean relative surprise across seeds was above the chance value of zero. For all five physical concepts, PLATO produced mean (M) relative surprise values above zero: continuity: M = 0.044, s.d. = 0.006, *t*(4) = 15.9, *P* = 4.6 × 10^−5^; directional inertia: M = 0.017, s.d. = 7 × 10^−4^, *t*(4) = 47.8, *P* = 5.7 × 10^−7^; object persistence: M = 0.034, s.d. = 0.008, *t*(4) = 8.7, *P* = 4.8 × 10^−4^; solidity: M = 0.009, s.d. = 0.003, *t*(4) = 6.4, *P* = 0.002; unchangeableness: M = 0.007, s.d. = 2.2 × 10^−4^, *t*(4) = 60.57, *P* = 2.2 × 10^−7^. Additionally, we performed a one-tailed, single-sample *t*-test to assess whether the average accuracy was above the chance value of 0.5. For all five physical concepts, PLATO produced accuracy values above 0.5: continuity: M = 0.891, s.d. = 0.028, *t*(4) = 27.7, *P* = 5 × 10^−6^; directional inertia: M = 0.727, s.d. = 0.017, *t*(4) = 26.9, *P* = 5.6 × 10^−6^; object persistence: M = 0.678, s.d. = 0.043, *t*(4) = 8.2, *P* = 5.9 × 10^−4^; solidity: M = 0.719, s.d. = 0.064, *t*(4) = 6.8, *P* = 0.001; unchangeableness: M = 0.656, s.d. = 0.021, *t*(4) = 14.7, *P* = 6.2 × 10^−5^. Furthermore, the time courses of relative surprise provided qualitative evidence that surprise rose at the moments in each probe video coinciding with the onset of the relevant physically impossible event (Fig. 5, bottom row).

In contrast, VoE effects were severely diminished or absent for the object-agnostic models (Fig. 5 top and middle rows, blue bars). The strongest results among the object-agnostic models came from the FEC baseline which was matched in representational capacity to PLATO (and thus contained many more free parameters). A one-tailed, single-sample *t*-test showed that the FEC object-agnostic model produced mean relative surprise above zero (Fig. 5 top row, blue bar) for only three physical concepts: continuity: M = 0.009, s.d. = 0.008, *t*(4) = 2.4, *P* = 0.038; directional inertia: M = 0.012, s.d. = 0.01, *t*(4) = 2.4, *P* = 0.036; object persistence: M = −3.9 × 10^−5^, s.d. = 6.4 × 10^−5^, *t*(4) = −1.2, *P* = 0.86; solidity: M = 9.3 × 10^−5^, s.d. = 2 × 10^−4^, *t*(4) = 0.92, *P* = 0.21; and unchangeableness: M = 1.4 × 10^−4^, s.d. = 1.3 × 10^−4^, *t*(4)=2.15, *P* = 0.049. A one-tailed, single-sample *t*-test showed that the FEC model produced accuracy scores (Fig. 5 middle row, blue bar) above zero for only two physical concepts: continuity: M = 0.71, s.d. = 0.15, *t*(4) = 2.8, *P* = 0.024; directional inertia: M = 0.69, s.d. = 0.2, *t*(4) = 1.9, *P* = 0.065; object persistence: M = 0.51, s.d. = 0.016, *t*(4) = 1.1, *P* = 0.168; solidity: M = 0.5, s.d. = 0.036, *t*(4) = 0.174, *P* = 0.435; and unchangeableness: M = 0.493, s.d. = 0.115, *t*(4) = −0.129, *P* = 0.548.

Taken together, these results indicate a strong facilitative role for object-level representation in the acquisition of intuitive physics concepts, consistent with the conclusions of developmental psychology literature.

#### Effect of training set size

**Fig. 6:**
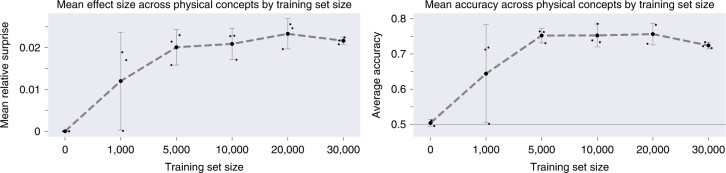
PLATO displays robust effects with as little as 28 h of visual experience. PLATO shows robust VoE effects (left) and accuracy (right) when the dynamics predictor trained with as little as 50,000 examples (28 h of visual experience). Training size = ‘0’ indicates no training at all, shows that object representations are not sufficient for VOE effects and that training the dynamics predictor is critical. Performance was averaged across all five physical concepts. Small dots depict different random seeds. Large dots depict mean over three seeds, with corresponding error bars showing 95% confidence intervals over seeds. Performance across seeds was assumed to be normal, but this was not formally tested.

The training corpus in the Physical Concepts dataset contains a total of 300,000 videos. By a conservative calculation (Methods), this adds up to approximately 52 d worth of continuous visual experience. It is a question of obvious interest, both from an AI and a developmental point of view, how much training data is actually required to yield VoE effects at test. To assess this, we trained three random seeds of PLATO’s dynamics predictor on datasets of gradually decreasing size (Fig. 6), calculating the ‘grand mean’ of VoE effects over all five probe categories (see Supplementary Fig. 12 for metrics on individual probe categories). After training on only 50,000 examples, a one-tailed, single-sample *t*-test revealed the grand mean relative surprise was above zero (M = 0.02, s.d. = 0.003, *t*(2) = 9.246, *P* = 0.006). Similarly, after 50,000 examples we found that the grand mean of PLATO’s accuracy scores was above 0.5 (M = 0.75, s.d. = 0.015, *t*(2) = 23.3, *P* = 9.2 × 10^−4^). These results indicate that robust VoE effects arise in our model after training with as few as 50,000 examples, the equivalent of 28 h of visual experience.

#### Generalization to unseen objects and events

**Fig. 7:**
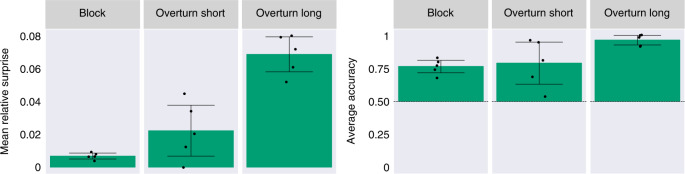
PLATO displays robust effects on unseen objects and dynamics without any retraining. PLATO displays robust VoE effects (left) on unseen objects and dynamics without any retraining. We use videos from three probe types in the ADEPT dataset to assess PLATO’s generalization capabilities. ‘Block’ is designed to test the concepts of solidity and continuity. ‘Overturn short’ and ‘Overturn long’ test the concepts of object permanence. We find reliable VoE effects in both of our metrics. The mean relative surprise (left) varies by probe type but is always above zero, indicating that impossible events are generally more surprising than their possible counterparts. Furthermore, the average accuracy (right) is well above chance (50%, dashed line) for all probe types. Each point represents the average performance over probe pairs for models trained (on our dataset, not ADEPT) with a different random seed. Error bars show the 95% confidence intervals over five seeds. Performance across seeds was assumed to be normal, but this was not formally tested.

As a strong test of generalization, we evaluated our model on object shapes and dynamics different from those presented during training. To do so, we leveraged the ADEPT dataset^29^, an independently developed, procedurally generated dataset designed to probe intuitive physics knowledge. As detailed under Methods, three probe types from the ADEPT dataset were amenable to the test procedure used in our main simulations. Labelled by their creators ‘overturn short’, ‘overturn long’ and ‘block,’ these probe types include object types not included in our own dataset (for example, rabbits and bowling pins), as well as novel movement patterns (a drawbridge-like plank that rotates not only down but also up). The first two probe types test object permanence using a rotating drawbridge exactly like Baillargeon’s original study^13^. The third probe tests the concepts of solidity and continuity using a design in a different developmental study^11^: a rolling ball approaches a wall that should stop it, is briefly occluded before contact, and then is revealed to lie on the opposite side of the wall. This ‘magic trick’ can be viewed as a violation of the principle of solidity (the ball rolled through the wall) or of continuity (the ball teleports to the other side of the wall). We tested PLATO on these probes without retraining or fine-tuning any part of the model, measuring prediction errors for impossible relative to possible probes, as in our original experiments. As shown in Fig. 7, PLATO displayed clear VoE effects for all three probe classes. A one-tailed, single-sided *t*-test showed that the mean relative surprise was above zero for all three probes: block: M = 0.007, s.d. = 0.002, *t*(4) = 7.5, *P* = 8.4 × 10^−4^; overturn long: M = 0.069, s.d. = 0.011, *t*(4) = 12.671, *P* = 1.1 × 10^−4^; overturn short: M = 0.022, s.d. = 0.016, *t*(4) = 2.8, *P* = 0.024. Similarly, PLATO’s accuracy was above 0.5 for all three probes: block: M = 0.765, s.d. = 0.049, *t*(4) = 10.9, *P* = 2 × 10^−4^; overturn long: M = 0.97, s.d. = 0.037, *t*(4) = 25.3, *P* = 7.2 × 10^−6^; overturn short: M = 0.79, s.d. = 0.16, *t*(4) = 3.56, *P* = 0.012.

## Discussion

‘Common sense’ reasoning, in numerous varieties, has been an increasingly pressing objective for AI research. In the present work, we reported progress toward building deep-learning systems that construct, on the basis of perceptual experience, knowledge in a specific but fundamental common-sense domain: intuitive physics. In doing so, we drew inspiration from developmental psychology on two fronts. First, to measure progress systematically and quantitatively, we ported the VoE paradigm from developmental psychology to probe five different physical concepts. Second, to build a model capable of learning intuitive physics, we endowed our model with object-centric representation and computation directly inspired by accounts of infant intuitive physics. To train that model, we created a training dataset of composable physical interactions yielding complex three-dimensional (3D) scenes, the Physical Concepts dataset (also making this freely available online as a resource for other researchers). To test the utility of the object-based approach, we compared our model with tightly controlled, object-agnostic baselines. Finally, to test the generalization capabilities of our network, we evaluated our model on an externally defined dataset with novel appearances, shapes and dynamics without any retraining.

Our results centre on four key observations. First, our object-based model displayed robust VoE effects across all five concepts we studied, despite having been trained on video data in which the specific probe events did not occur. Second, consistent with expectations founded on the developmental literature, we found that the VoE effects seen in our model diminished or disappeared in well-matched models that did not employ object-centred representations. Third, we observed that robust VoE effects could be obtained with as little as 28 h of visual training data. Finally, we report that our model generalized to an independently developed dataset involving novel object shapes and dynamics.

Our work relates closely to, and also substantially extends, a number of previous studies in AI and computational cognitive science. Two other groups have built intuitive physics evaluation frameworks specifically using the VoE paradigm. Riochet et al.^28^, in research conducted in parallel with our own original work in this area^27^, presented a dataset for probing a variety of physical concepts, including a subset of those covered in our Physical Concepts dataset. Although the videos comprising this dataset include richer textures and lighting effects than our dataset, the physical events themselves are more restricted in their diversity. Smith and colleagues^29^ introduced the ADEPT VoE dataset mentioned earlier, which is still simpler in design and eschews collision events. Neither of these preceding studies reported a system that successfully acquires specific physical concepts through learning: the model presented by Riochet and colleagues showed VoE effects only in a minority of conceptual categories tested, and the model presented by Smith and colleagues embeds a hand-engineered physics engine rather than learning physics from scratch.

Our work extends these previous work by introducing a new dataset involving a more dramatic separation between training and test events, combined with object dynamics more faithful to physics. Of course, despite these advances, the range of object and event types in our dataset remains narrow compared with those encountered in the real world. Increasing richness and ecological validity while also maintaining experimental control is a challenge in AI just as it is in psychology, but growing research of the present kind towards richer data domains is of course an important aspiration. Fortunately, the architecture we have introduced is neither tied to the particular object types and events contained in our dataset, as demonstrated in our ADEPT experiment, nor is there an a priori reason to expect that a similar computational approach cannot be extended to more naturalistic event types. Indeed, relational architectures related to PLATO have recently been employed in engineering research to learn the dynamics of quite complex physical systems^38^.

In presenting our approach, we have stressed the centrality of learning. In view of this, it is important to acknowledge that our model implementation was granted access to two sources of privileged information: object (segmentation) masks and object indices allowing consistent placement of object embeddings within the model over timesteps (tracking). Recent research has introduced methods for object segmentation and tracking that would allow each of these to be extracted from pure video data without access to privileged information of any kind. In the case of object masks, several recent papers have introduced methods for unsupervised object discovery (see refs. ^39,40^ for example). Recently, methods for learning to track objects consistently over time have been reported^41,42^. Integrating these methods into our framework to allow true ‘learning from scratch’ represents an appealing goal for next-step development. In the meantime, it is worth noting that the mere availability of object-level segmentation and tracking information does not fully explain the simulation results we have presented. In our training-set size analysis (Fig. 6), the model without any training at all (‘0’) failed to produce any VoE effects. In a related simulation work also focusing on VoE effect in intuitive physics, a model that was given segmentation masks but which did not carry out relational object-level processing failed to produce VoE effects^28^.

The advantages of object-level representation observed in the present work echo related findings from other areas of AI research, including question-answering^43^ and reinforcement learning^20,44–47^, where object-level representation has been found to support faster learning and improve transfer. What is the explanation for the benefit of object-level representation in the problem setting we have studied? We speculate that the advantage derives from the role that object-level representation can play as a regularizer. In a machine-learning context, regularization refers to the inclusion of constraints on a learning process, which bias that process to place greater weight on particular inferences from training data^48^. Regularization, in this sense, is employed to prevent a learning system from ‘overfitting’ to training data, learning representations that are so closely tied to the specifics of that data that the system fails to identify more general regularities that would allow it to perform correctly on new data at test. A bias towards object-level representation could have this kind of regularizing impact on visual learning, in the sense that it biases the learning system towards a compositional representation of visual events which corresponds to the actual compositional structure of the physical events themselves, resulting in knowledge that generalizes well to new events. While this explanation is speculative, it aligns with one informal observation from preliminary modelling work not described above. In this preliminary modelling, we trained object-based and flat models on videos in which the depicted events were much closer in form to those presented at test in the VoE probes. Under these conditions, the difference in VoE effects between object-based and flat models was considerably smaller than what we observed in the simulations that we report in the present paper, which featured much greater separation between training and testing data. It is in this setting, where a learning system must transfer at test to data that lies reasonably far from any particular training example, that regularization can be decisive. Not only does this regularization have the potential to make an intractable task tractable, but it may also make models more data-efficient. Where deep-learning models have often been criticized as being too data inefficient^5^, our results demonstrate that a relatively small amount of visual experience—on the order of tens of hours—is sufficient to engender robust VoE effects in response to violations of physics. Exploring this regularization-based interpretation of the role of object-based representation thus stands as an appealing hypothesis for further investigation. In this connection, it is worth noting that recent work in AI has increasingly favoured computational architectures (for example, graph nets and tranformers) that implement an inductive bias towards relational, compositional processing^49^.

Throughout the present work, we have emphasized the role of insights from developmental psychology in guiding the research we have reported. What, if any, are the implications of our work for developmental psychology itself? This topic must be approached with some care, since the model that we have presented is not intended to provide a direct model of physical-concept acquisition in children. Nevertheless, we do think there are several insights that may be germane to developmental science. First, our modelling work provides a proof-of-concept demonstration that at least some central concepts in intuitive physics can be acquired through visual learning. Although research in some precocial species suggests that certain basic physical concepts can be present from birth^50^, in humans the data suggest that intuitive physics knowledge emerges early in life^31^ but can be impacted by visual experience^51,52^. Of course there is extensive debate and legitimate uncertainty about innateness^11,53^. Our modelling work complements these conclusions from experimental research by demonstrating the sufficiency of visual learning to explain the emergence of one core set of physical concepts, assuming—as developmental psychology led us to anticipate—that object-level representations are available. Furthermore, our model reflects just one point in a family of possible models that implement proposed algorithmic-level^54^ principles (for example, tracked objects) suggested by developmental psychologists. Exploring different ways of implementing these principles (for example, replacing our object buffer with a recurrent object tracker or removing recurrence from the dynamics predictor; see Supplementary Fig. 12) presents a natural avenue for future work to make more precise contact with developmental accounts at the implementation level^54^.

It would be fascinating to extend the present modelling work to make even more direct contact with key questions in developmental psychology. For example, the order of concept acquisition throughout development has been of central interest to developmental psychology^55^. Although we do not model developmental trajectories in the present work, there is a long history of using connectionist models to shed light on stage-like developmental processes, including in the domain of intuitive physics^56^. Recent work^57^ is extending the VoE effect to neurophysiological measurements, potentially opening up new possibilities for testing and constraining models of the kind we have proposed here. Bringing the present work to bear on these questions poses an obvious research opportunity. This would be especially promising in combination with newly emerging datasets that capture first-person footage of infant experience at large scale^58,59^. This represents just one opportunity that our model might offer as a tool for computational research into the origins of intuitive physics in human development. A broader range of possibilities follows from the wider class of relational AI systems mentioned briefly above^49^. Whereas the present work has explored the utility of relational, object-based processing for understanding intuitive physics, we speculate that relational mechanisms currently being explored in AI may be useful for understanding human knowledge in other ‘core domains’ studied in developmental psychology^12^, perhaps most interestingly the interactions with animate agents.

## Methods

### Training dataset, ‘freeform’ physical events

Our training dataset consists of 300,000 procedurally generated scenes using custom Python code and the Mujoco physics engine. Our motivation was to build a dataset for training that encompassed a wide range of complex physical interactions. Thus, we built composable scene building blocks: rolling, collisions along the ground plane, collisions from throwing or dropping an object, occlusions (via a ‘curtain’ that descends from the top of the screen and retracts), object stacks, covering interactions (an open-bottom, closed-top container falls onto an object), containment events (an object falls into an open-top container) and rolling up/down ramps. Each building block used randomly generated values for object appearances and locations, while ensuring the main intended physical interaction still occurred.

To form a scene, we chose two to four scene building blocks. Building blocks compose by advertising and/or consuming locations of interest. For example, ‘rolling’ building blocks have two locations of interest: the rolling object’s starting point and the point it rolls towards. An ‘object stack’ building block has a single location of interest: the centre of the object stack. Composing these two building blocks can yield a scene where a sphere rolls at the object stack. Alternatively, the sphere can start off at the top of the object stack and roll off of it. With some probability, building blocks do not compose and inhabit the same scene independently.

We restricted the primitive shapes in our dataset to rectangular prisms and spheres. From the rectangular prisms we built a ‘curtain,’ a ramp, an arch, and both open-top and closed-top containers. Object sizes were chosen in a hand-crafted range to ensure that objects were visible. Object colours and the checkerboard floor were chosen as random red-green-blue (RGB) values. Objects had varying but stereotyped masses. Rolled objects and objects in an object stack had a mass of 10. Dropped or thrown objects were made four times heavier so that they could more easily displace objects they hit. Containers had a mass of 4 or 5. Arches had a total mass of 60 to keep them upright. We did not withhold any object shapes from the training data for use in the test probes. Learning to generalize the dynamics of arbitrary shapes would be its own research programme with significant challenges. We could sidestep this issue by creating probes that employ novel shapes but endow them with familiar dynamics: a chess piece that glides across a frictionless floor would have the same lateral trajectory as one of our rolling balls. However, this would be a contrived version of physics that could only provide a superficial test of generalization to new objects. Each scene unfolded over 3,000 simulation steps and was rendered into 15 frames at a resolution of 64 × 64 RGB pixels. Each frame had a corresponding segmentation mask rendered at the same resolution where each object in the scene, including the floor, was given a unique and consistent value. To add extra complexity, we used a drifting camera. The position and orientation of the camera was the same at the start of all scenes. From there the camera randomly drifted its position and location (subject to a bounding box to ensure the camera was still looking at relevant parts of the scene). For each of the 15 frames in a video, we exported the camera position and orientation and gave this information to our model. We chose a checkered background to provide a depth cue for the relative positions of objects as they and the camera position moved throughout a video. We added this for visual diversity and to make our results comparable to that of ref. ^27^. Finally, we built an additional 5,000 ‘freeform’ scenes with this same structure to use as a test set during hyperparameter selection.

### VoE paradigm

As described earlier, to assess the model’s knowledge of specific physical concepts, we leverage the VoE paradigm from the developmental literature and adapted for artificial models^27,28^ in parallel and subsequently^29^. We generated 5,000 probe scenarios for each of the following physical concepts adapted from developmental psychology: object persistence, continuity, unchangeableness, directional inertia and solidity (described below). Whereas the training dataset had a freely moving camera, we used a stationary camera for the probes to ensure objects were (where applicable) occluded during the transition from physical to aphysical frames. We computed surprise as the model’s prediction errors over the course of a video. Although we optimized the loss in the object code space, for evaluation we looked at the error in pixel space. We can take this error in pixel space by decoding object codes to images using the ComponentVAE’s decoder, Θ.

$$surprise(video)=\mathop{\sum }\limits_{t=1}^{T-1}\mathop{\sum }\limits_{k=1}^{K}{({{\Theta }}({z}_{k}^{t+1})-{{\Theta }}({\hat{z}}_{k}^{t+1}))}^{2}.$$

we took this loss in pixel space, instead of in object code space which we used for training, due to the high variance of object codes representing empty ‘objects’. This arises because the ComponentVAE emits a fixed number (*K* = 8) of output components (‘objects’). If there are fewer than 8 objects in the image, then the ComponentVAE outputs ‘objects’ which correspond to blank pixels. As these empty objects were fairly prevalent in our scene (most videos did not contain the maximum of 8 objects in a scene), the learned representational code uses the unit Gaussian (mean = 0, variance = 1) to represent empty components. To represent components with actual objects in them, the ComponentVAE shifts the mean from 0 and decreases the variance (Supplementary Fig. 18). Because of the high variance on empty objects, it is relatively difficult to predict the exact value of an empty slot across timesteps. By taking pixel loss, we avoided this issue as any of the values that encode an empty object all decode to a blank image. Furthermore, because the possible and impossible videos within a probe scenario contained the same objects, we avoided issues that typically arise when using pixel loss. For example, in pixel space, predictions of large objects carry more weight than predictions of small objects, but within a probe scenario the objects in the possible and impossible videos are perfectly matched.

### Physical concept ‘object persistence’

Perhaps the most fundamental aspect of intuitive physics is understanding that objects cannot disappear from existence. This is referred to as ‘object permanence’. The principle of ‘object persistence’^10^ extends this to say that “objects persist, as they are, in time and space”. Taking inspiration from a classic behavioural experiment^13^ on object permanence, probes for this category involved a rigid plank falling on an object. In the possible probe (Supplementary Fig. 1), when the plank fell on the object, the plank occluded it while also remaining propped up by it as expected. By contrast, in the impossible probe the plank fell on top of the object (in a manner that is initially identical) but ended up flat on the floor, as if the object had disappeared. In the counterbalanced probes, the possible probe had the plank falling flat on the floor in an otherwise empty scene, and the impossible probe had the plank falling in the same empty scene but ended up inexplicably propped up by an item that was made to appear under the plank while it occluded part of the floor. We flagged this as a test of object persistence because not only must the object continue to exist while being occluded, but it must also retain its properties. For example, if the object shrank while occluded, then the plank could occupy the space it occupied without violating physics.

### Physical concept ‘unchangeableness’

By the principle of ‘unchangeableness’^9^, objects tend to retain their features (for example, colour, shape) over time. In the possible probes of this dataset (Supplementary Fig. 3), a random assortment of static objects were aligned in the foreground. A screen was lowered in front of those objects, and was then raised.

The concept of ‘unchangeableness’ relates to a number of different aspects of objects, and therefore in the impossible probes we swapped the positions of objects when they were behind the curtain to suggest that their position, colour or shape had changed.

The developmental study that inspired our event design^30^ used separate occluders for each object. To simplify procedural event generation, our probe videos involved a single occluder spanning all objects. It should be noted that, in contrast to the developmental study, the ‘impossible’ events in our videos were therefore susceptible to an alternative interpretation, whereby objects traded places through self-propelled motion. In the developmental literature, this would be classified not as a violation of unchangeableness, but instead as a detection of (implausible) self-propelled motion in animate objects^60^.

### Physical concept ‘continuity’

The concept that an object traces out one continuous path through space and time is referred to in the developmental literature as ‘continuity’^31^. For videos (Fig. 1) in this category, we used a nearly identical setup to a classic experiment^26^ where possible probes began with two static pillars separated by a gap. A ball was rolled horizontally behind both pillars so that it was visible before, between and after the pillars during its trajectory. In the impossible probes, while the ball was occluded by the first pillar, the ball was made invisible for the period when it would be between the pillars, and then reappeared after the second pillar. Because the size of the pillars matched the size of the ball, it was not possible that the ball was simply stopped behind the first pillar. Alternatively, we made the ball only visible when it was rolling between the pillars, but not before or after. The only way this could be possible would be if there were self-propelled balls, which never appeared in our dataset. Note that the splicing procedure for probes for this concept deviated slightly from what is shown in Fig. 4. However, we still maintained the image-level and pairwise matching of frames across possible and impossible videos in a probe tuple.

### Physical concept ‘solidity’

This dataset is an adaptation of an experiment that uses an object and an occluder^22^ to test understanding of the solidity of objects, as related to the penetration of an object through a container and the ground below. Although originally situated in an analysis of the differences between infant cognition in occlusion versus containment events, it nonetheless relies on the concept of solidity. In probes, perspective was carefully controlled such that the camera can view inside the top of the container but not the bottom. In possible probes (Supplementary Fig. 2), a rectangular block was dropped into the container and came to rest as expected. In the impossible probes, the object ‘fell through’ the container and the floor, and therefore disappeared from view (with the penetration itself occluded by the face of the container) even if the object should have remained visible due to its height. To allow for the bias-free splicing procedure, we had an alternate impossible probe condition which related to solidity in a different way: the object remained visible at the top of the container when its height clearly dictated that it should have fallen further into the container. In this case, the violation is that objects can only rest upon solid surfaces. In terms of developmental theory, this relates closely to what have been termed the inertia and gravity principles^61^.

### Physical concept ‘directional inertia’

This dataset is a loose adaptation of a paradigm developed by Spelke et al.^62^ for investigating infant knowledge of inertia. Where the classic paradigm investigated both magnitude and directional violations of the principle of inertia under occlusion, the current probe only tests the directional component in an unoccluded fashion. The altered version was chosen to allow for the bias-free splicing procedure. Furthermore, we wanted to include a test involving collisions (which is a common, albeit in 2D not 3D, domain for learning physical dynamics with deep learning; for example, ref. ^63^). Possible probes (Supplementary Fig. 4) were formed by rolling a ball at an angle towards a heavy block. Upon contact, the sphere rolled away from the block while reflecting its velocity about the angle of incidence (as expected). The paired possible probe reversed this trajectory: it began at the end point of the first possible probe, rolled at the same location on the block and bounced off the block to end up at the initial position of the first probe. Each impossible probe was formed by swapping the trajectories of the impossible probes at the point where they made contact with the block. The effect of this swap was that when the ball hit the block, instead of reflecting about the angle of incidence, it headed back towards its initial location, clearly violating the principles of directional inertia for colliding objects.

### Model architecture

To implement our object-centric approach, our model used two main components: a feedforward perceptual module and a recurrent dynamics predictor.

The perceptual module took as input a 64 × 64 RGB image and 64 × 64 segmentation mask consisting of *n* = 8 channels and produced an object code for each (potentially empty) channel in the segmentation mask through unsupervised representation learning. Although not provided as inputs or targets to the perceptual module, we found that some of the axes of the code represented interpretable, static properties of the objects such as position on the ground plane or above the ground, height, width and different types of shape transformations (see Figs. 19–23 in Supplementary Material for traversals of this learned latent space). These object codes were fed into the dynamics predictor, which was then tasked with predicting the object codes at the next timestep. Instead of directly predicting the next image in the video, the dynamics model predicted per-object percepts.

### Perception module

To learn these object codes, we used ComponentVAE^39^. We used a convolutional neural network for the encoder Φ and a spatial broadcast decoder θ^64^. Where that work learned segmentation masks using an attention network, we used ground truth segmentation masks from the dataset. For a given image *x* and a segmentation mask *m* with *K* = 8 channels (representing a maximum of 8 objects in any scene), the ComponentVAE yielded *K* 16-dimensional Gaussian posterior distributions *q*_*ϕ*_(*z*_*k*_∣*x*_*k*_, *m*_*k*_) (where *x*_*k*_ is the masked image). The sample from this code, *z*_*k*_, was the object code for the *k*th object. The decoder took as input *z*_*k*_ to reconstruct masked object image and mask: ${p}_{\theta }({\hat{x}}_{k},{\hat{m}}_{k}| {z}_{k})$. Note that similar to ref. ^39^, we treated the ground plane as its own object with its own set of parameters in the encoder and decoder. Additionally, we always output 8 codes that were used by the dynamics predictor. Where there were fewer than 8 objects in the image (some of the mask channels were empty), the ‘extra’ slots were tasked with reconstructing an empty object image and mask.

During an offline phase, before training the dynamics predictor, we optimized the variational objective as defined by ref. ^39^ to learn the encoder and decoder parameters. We set *β* to 0.1 and *γ* to 10 to ensure that the model reconstructed the object masks with high fidelity. We found that even at this relatively low value of *β*, the model learned disentangled representations^65^ for each object, sometimes corresponding to factors such as position, shape, colour and size (see Figs. 19–23 in the Supplementary Information).

We used the pretrained ComponentVAE in two ways during the prediction task. First, to actually obtain the object codes, we fed in a segmented image into the ComponentVAE encoder to get the per-object posterior distributions. The object code was then obtained by taking a sample from each posterior distribution. Although it might seem more natural to use the mean instead of a sample, we found better results using the sample on our training data. Thus, each object code was a 16-dimensional vector in a learned representational space. Second, we used the ComponentVAE’s decoder to map the object codes predicted by our model to per-object images (which were easily composed to form a composite image). This allowed us to visualize the model’s predictions.

### Dynamics predictor

Where the perception module captured static object properties, our dynamics predictor needed to integrate information over time (for example, to calculate velocities or remember occluded objects). Thus, we employed a recurrent module for the dynamics predictor. We developed the ComponentLSTM, which is an objectwise LSTM (with 2,056 hidden units) with shared weights *ψ*, but object-specific activations. At each timestep, the LSTM for the *k*th object computes the following:

$${\hat{z}}_{k}^{t+1},\mathrm{cel{l}}_{k}^{t},\mathrm{hidde{n}}_{k}^{t}=\mathrm{LSTM}({z}_{k}^{1:t},\mathrm{cel{l}}_{k}^{t-1},\mathrm{hidde{n}}_{k}^{t-1};\psi ),$$

where the LSTM function is shorthand for the standard LSTM^66^ updates and ${\hat{z}}_{k}^{t+1}$ is the model’s prediction for object code *k* in the next image. The ${z}_{k}^{1:t}$ here is the object buffer: it is the set of all previously seen object codes for the *k*th object. Although in principle we could feed in just the the most recent object codes, we found much better results on our training data when using the full history (see ‘Hyperparameter Selection’ in Supplementary Information for motivation on this and other model hyperparameter choices). Because the segmentation masks were consistently ordered throughout the course of a video, the perceptual module’s outputs were also in a consistent ordering. Thus, when we aggregated object codes into the object buffer and fed them into the corresponding slot in the LSTM to make a prediction for the corresponding object, we endowed our model with object tracking.

Thus far, we have not equipped the dynamics predictor with a mechanism for computing the influence of objects on each other. To do so, we leveraged an Interaction Network^63^. We computed pairwise interactions from the LSTM cell states to both the cell states (using multi-layer perception (MLP_*ρ*_)) and the object buffer inputs (using MLP_*λ*_). Each MLP consisted of three layers with 512 units and the Gaussian Error Linear Unit activation function^67^. Briefly (see ‘Computing Interactions’ in Supplementary Information for details), for the *kth* memory slot in the LSTM, $\mathrm{cel{l}}_{k}^{t-1}$, we compute:

$$\mathrm{in{t}}_{k}=\mathrm{InteractionNetwork}(\mathrm{from}=\mathrm{cel{l}}_{k}^{t-1},\mathrm{to}=[\mathrm{cel{l}}_{1:K}^{t-1};{z}_{1:K}^{1:t}]).$$

To aggregate the interactions across all pairs for the *k*th slot, we computed the sum and the maximum of the interactions and concatenated these values together. We added the corresponding interactions as an additional input to the *k*th LSTM, yielding the final update computation:

$${\hat{z}}_{k}^{t+1},\mathrm{cel{l}}_{k}^{t},\mathrm{hidde{n}}_{k}^{t}=\mathrm{LSTM}(\mathrm{concat}({z}_{k}^{1:t},\mathrm{in{t}}_{k}),\mathrm{cel{l}}_{k}^{t-1},\mathrm{hidde{n}}_{k}^{t-1};\psi ).$$

we call the combination of an InteractionNetwork and ComponentLSTM an ‘InteractionLSTM’. Additionally, we included a residual connection from the *k*th object code for the current timestep ${z}_{k}^{t}$ to the model’s prediction for the *k*th object (once again exploiting aligned/tracked objects).

Finally, recall that the training data included a randomly drifting camera. Thus, we fed the ‘InteractionLSTM’ with viewpoint information as additional inputs. First, the interaction network received information about the current camera position. Additionally, the ComponentLSTM received information about the camera position at the next timestep. In this way, the dynamics predictor was not only tasked with predicting dynamics, but also predicting dynamics from a specific viewpoint.

We trained the dynamics predictor as a next-step predictor on the sequence of *T* = 15 VAE-compressed images for each video using teacher-forcing. At each timestep *t*, we converted an input segmented image to a set of object codes ${z}_{1:K}^{t}$, using the perceptual module’s encoder. We formed the object buffer ${z}_{1:K}^{1:t}$ by concatenating these object codes for all previously seen images from the video. To form a fixed-length vector across the sequence, we padded the object histories with zeros where necessary. Before feeding the object buffer into any downstream computations, we projected them into a 1,680-dimensional space using the Exponential Linear Unit activation function^68^. Camera information and the object buffer were fed into the InteractionLSTM to yield object code predictions ${\hat{z}}_{1:K}^{t+1}$. We optimized the sum of the mean-squared error between our predictions and the observed object codes at the next timestep:

$${{{{\mathcal{L}}}}}_{\mathrm{objectcode}}=\mathop{\sum }\limits_{t = 1}^{T-1}\mathop{\sum }\limits_{k = 1}^{K}{({z}_{k}^{t+1}-{\hat{z}}_{k}^{t+1})}^{2}$$

### Baseline models

Where PLATO used a slotted perceptual module—each slot encoded a distinct object—the flat baseline models encoded the entire scene in a single slot. This translated naturally into the dynamics predictor which made predictions based off of a single slot. The updates in the flat models were identical to the PLATO update equations above, with the exception that *K* = 1 instead of *K* = 8. Because there was only one slot, this meant that the flat module did not get any information in the segmentation mask (it had only a single channel and was uniformly filled to 1). However, see ref. ^28^ for evidence that non-object-based models trained with segmentation masks still fail intuitive physics examinations.

### Experiments

#### Training

To train the perception module, we used the RMSProp optimizer, with a learning rate of 1 × 10^−4^ for 1,000,000 steps with a batch size of 64 images. To train the dynamics predictor, we trained our models for a total of 1,300,000 training steps with a batch size of 128 videos. We used a learning rate that transitioned from 1 × 10^−4^ to 4 × 10^−5^ after 300,000 training steps. The above training procedure was used for all models and all experiments.

#### PLATO vs flat models

For each model, we pretrained the perception module on the full dataset of 4,500,000 images (300,000 videos each with 15 frames of content). We then trained the dynamics predictor, freezing the weights of the perception module, using five different random seeds for weight initialization. For each seed of the dynamics predictor, we ran 5,000 probe quadruplets for each of the five concepts.

#### Training set size

As above, we pretrained a perceptual module on the full dataset size of 300,000 videos (4,500,000 images). We then varied the dataset size used to train the dynamics predictor using three random seeds while freezing the weights of the perception module. Training the dynamics predictor proceeded as above regardless of dataset size. We also included a ‘0’ training set size which corresponded to a model with an untrained dynamics predictor. This model still received object percepts from the pretrained perceptual module. The lack of VoE effects for the ‘0’ model shows that the VoE effects depend on more than just tracked object representations. Predicting dynamics is necessary for VoE effects on the five physical concepts probed.

### Visual experience calculation

To compute the visual experience contained in our dataset, we calculated the following: 300,000 videos each lasting 2 s yielded 600,000 s or roughly 6.95 d of continuous visual experience. For only 50,000 videos, this amounts to roughly 28 h of continuous experience. Conservatively assuming 8 h of wakeful experience in a day yields an equivalent of roughly 20.9 d for 300,000 videos or 3.5 d for 50,000 videos.

### Generalization test using ADEPT dataset

To measure our model’s ability to generalize, we tested PLATO on videos mined from the ADEPT dataset^29^. The dataset contains procedurally generated probes (scenarios in ADEPT) of intuitive physics, with objects entirely different from those of our own dataset. Each example in the dataset contains RGB images and corresponding object masks. The probes are organized into eight types. Similar to our probe quadruplets, ADEPT provides sets of possible and impossible videos for each probe type, although they do not use the same splicing procedure to carefully control for image-level differences between possible and impossible videos.

We had to make two changes to the ADEPT probes to apply PLATO. The first class of changes was cosmetic: we applied cropping and downsampling to the videos to make the videos match PLATO’s expected input size. Second, we had to confront the fact that PLATO expects input masks to be in a consistent ordering throughout a video. However, ADEPT only provides aligned input masks for one probe type: overturn short. Through a hard-coded, type-specific procedure, we were able to manually align two additional probe types: overturn long and block. In this way, we were able to ‘mine’ three probe types from the ADEPT dataset that span the concepts of object permanence, solidity and continuity. This served as a useful measure of PLATO’s generalization capabilities, but importantly cannot be compared directly to the ADEPT results.

First, we applied superficial changes to match the image size and video length expected by PLATO. We centrally cropped the ADEPT dataset from 320 × 480 pixels to 320 × 320 pixels. We manually verified on a subset of probes that this still displayed the relevant parts of the probe displays. Subsequently, we downsampled from 320 × 320 pixels to 64 × 64 pixels to match PLATO’s expected input size. Finally, PLATO expects to operate over a video of 15 frames, but ADEPT had much higher temporal resolution and videos which varied in length by probe type. Thus, for each of the different probe types, we changed the downsampling factor to always yield 15 frames: block was downsampled by a factor of 12, overturn short by a factor of 8 and overturn long by a factor of 15. Again, we manually verified on a subset of examples that the downsampled videos retained relevant events of the possible and impossible probes.

We evaluated PLATO on our modified versions of the overturn short, overturn long and block probes without any retraining of our model. Where our dataset contained probe quadruplets (two physically possible videos and two physically impossible videos), ADEPT contains pairs of videos: one physically possible and one physically impossible. This allowed us to compute essentially the same metrics used for our dataset: mean relative surprise and average accuracy. The only difference was that we ‘summed’ over a single video instead of two videos. We performed this evaluation for five seeds of our model trained with our full training dataset to produce Fig. 7.

### Reporting summary

Further information on research design is available in the Nature Research Reporting Summary linked to this article.

## Supplementary information

Supplementary Information Supplementary Figs. 1–23.

Reporting Summary

Supplementary Video 1 This video explains various aspects of the ‘freeform’ data we generated to train our models.

Supplementary Video 2 We visualize next-step predictions for PLATO. At each frame of the visualization, we show PLATO’s prediction for the next frame on the basis of encoded input frames from the dataset.

Supplementary Video 3 We visualize ‘rollouts’ of PLATO’s predictions. Rollouts are generated by feeding PLATO-encoded input frames from the dataset. Halfway through the video, we start feeding PLATO’s outputs as inputs to simulate multi-step predictions.

## Data Availability

Instructions on accessing the physical concepts dataset are available at https://github.com/deepmind/physical_concepts

## Code availability

Our implementation of PLATO is not externally viable, but the corresponding author may be contacted for any clarifying questions on implementation details for building PLATO.
