# Publisher Correction: Intuitive physics learning in a deep-learning model inspired by developmental psychology

**DOI:** 10.1038/s41562-022-01432-5

**Published:** 2022-07-20

**Authors:** Luis S. Piloto, Ari Weinstein, Peter Battaglia, Matthew Botvinick

**Affiliations:** 1 DeepMind, London, UK; 2 Princeton Neuroscience Institute, Princeton University, Princeton, NJ USA; 3 Gatsby Computational Neuroscience Unit, University College London, London, UK

**Keywords:** Psychology, Cognitive neuroscience, Computational models, Philosophy

Correction to: *Nature Human Behaviour* 10.1038/s41562-022-01394-8, published online 11 July 2022.

In the version of this article initially published, in the sixth paragraph of the main text, the text now reading “In the present work, we introduce a much richer video corpus, the Physical Concepts dataset (https://github.com/deepmind/physical_concepts),” originally listed placeholder text instead of the dataset link. The error has been corrected in the HTML and PDF versions of the article.

