# Supplementary material for: Intuitive physics learning in a deep-learning model inspired by developmental psychology
Source: Nat Hum Behav. 2022 Jul 11;6(9):1257–67. doi: 10.1038/s41562-022-01394-8 (PMC9489531; doi:10.1038/s41562-022-01394-8)
Supplement: Supplementary file 1 — Supplementary Figs. 1–23. [file 41562_2022_1394_MOESM1_ESM.pdf]

nature portfolio

https://doi.org/10.1038/s41562-022-01394-8

Supplementary information

Intuitive physics learning in a deep-learning model inspired by developmental psychology

In the format provided by the
authors and unedited

Supplementary Information: Intuitive physics learning in a deep-learning model inspired by developmental psychology

Luis Piloto, Ari Weinstein, Peter Battaglia, Matthew Botvinick

Physical Concept Probes

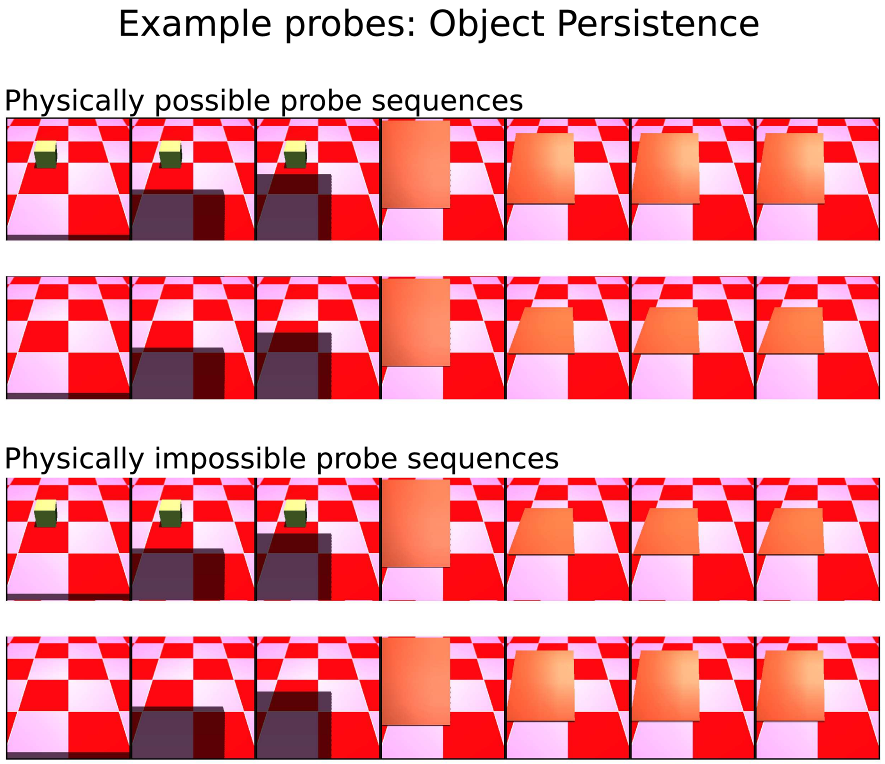

Supplementary Figure 1: Example probes adapted from (1) to assess the physical concept of object persistence (2). Each row corresponds to one temporally down-sampled video in a probe tuple. Actual videos consist of a total of 15 frames. The top two rows are physically possible probes and the bottom two rows are physically impossible probes. **Physically Possible Probes**: In the first physically possible probe (first row), a rigid plank falls onto, occludes, and is propped up by a cube. Note that the dark areas in the first three frames are the shadow of the falling plank, which is not yet directly in the field of view. In the second possible probe (second row), the plank falls and remains flush against the ground. **Physically Impossible Probes**: These probes are formed by splicing parts of the physically possible probes into impossible events. In the first impossible probe (third row), the plank falls onto the cube. However, instead of being propped up by the cube as you would expect, the plank ends up flush against the ground as if no cube existed at all. The second impossible probe (fourth row) shows a plank falling where there is no cube. Although it should end up flush against the ground, the plank comes to rest at an angle as if it landed on a cube.

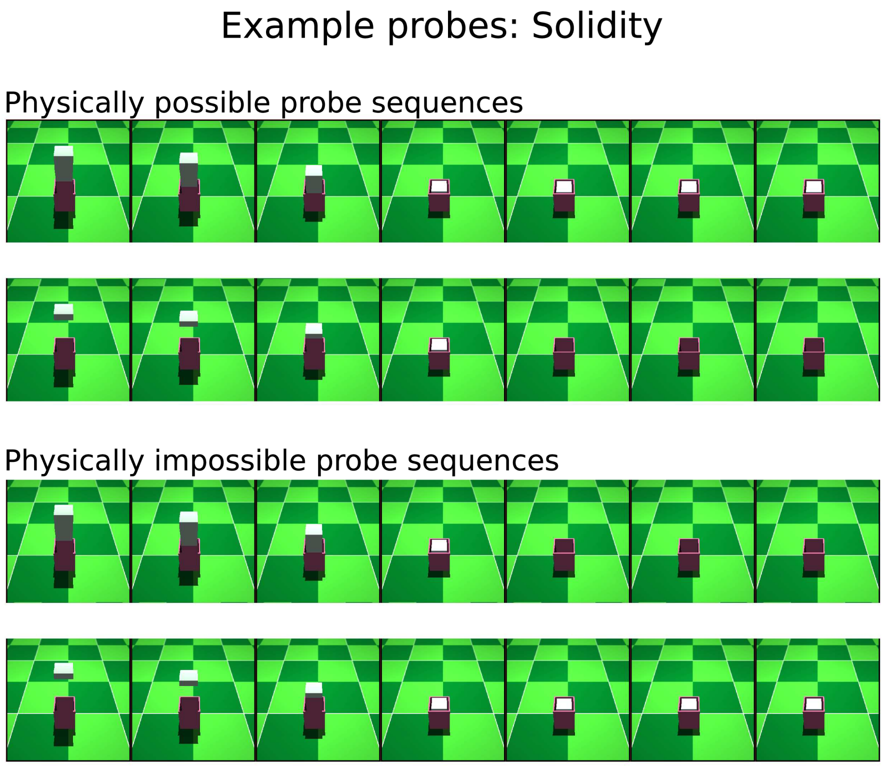

Supplementary Figure 2: Example probes adapted to assess the physical concept of solidity: objects do not interpenetrate. We take the design from (3), but use it to test the concept of solidity despite being initially developed to assess perception of occlusions versus containment events. Each row corresponds to one temporally down-sampled video in a probe tuple. Actual videos consist of a total of 15 frames. The top two rows are physically possible probes and the bottom two rows are physically impossible probes. **Physically Possible Probes**: In the first physically possible probe (first row), a tall block falls into a visibly empty container. In the second possible probe (second row), a short block falls into the container. **Physically Impossible Probes**: These probes are formed by splicing parts of the physically possible probes into impossible events. In the first impossible probe (third row), the tall block continues to fall into the container as if there was no bottom. In the second impossible probe (fourth row), the short block falls into the visibly empty container but fails to fall all the way in. See Methods for more detailed conceptual analysis.

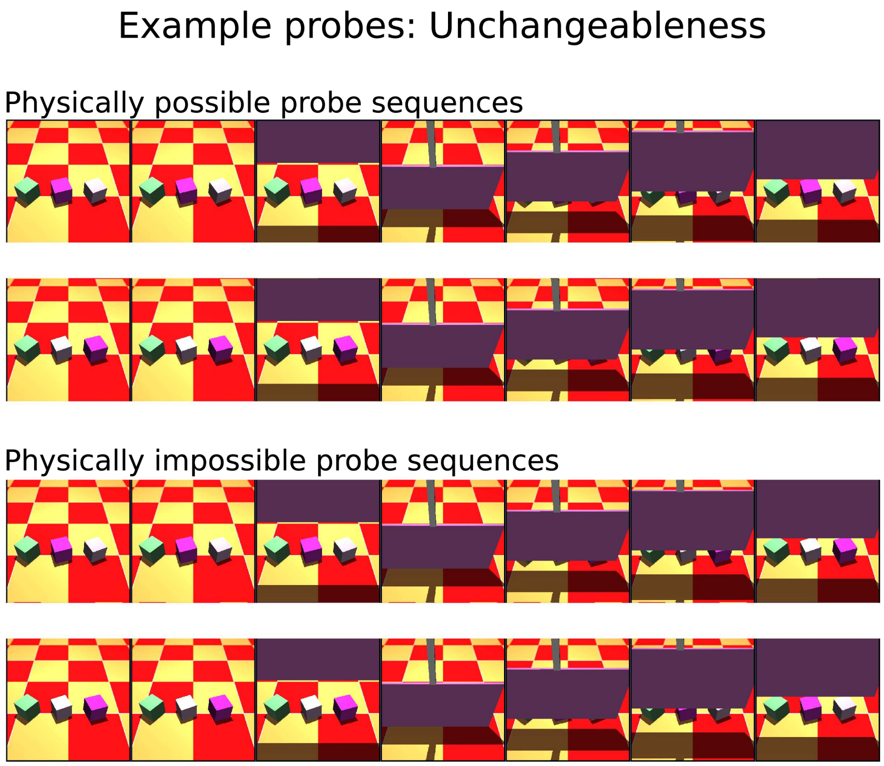

Supplementary Figure 3: Example probes adapted from (4) to assess the physical concept of unchangeableness (5; 6): objects have certain immutable attributes (e.g. they do not change shape, size, or color). Each row corresponds to one temporally down-sampled video in a probe tuple. Actual videos consist of a total of 15 frames. The top two rows are physically possible probes and the bottom two rows are physically impossible probes. **Physically Possible Probes**: In the first physically possible probe (first row), an arrangement of objects is occluded by a descending curtain and eventually unoccluded to reveal the initial configuration of objects. In the second possible probe (second row), the same exact objects appear in a different configuration. They are then occluded and unoccluded as in the first probe. **Physically Impossible Probes**: These probes are formed by splicing parts of the physically possible probes into impossible events. In the first impossible probe (third row), the objects begin in the configuration of the first probe. However, when the occluder is lifted, the objects are seen in the configuration of the second probe, suggesting that the objects have somehow changed shape, size, or color while occluded (note: the example above only depicts a color change). The second impossible probe (fourth row) starts with the configuration from the second possible probe and ends with the configuration from the first possible probe. See Methods for further conceptual analysis.

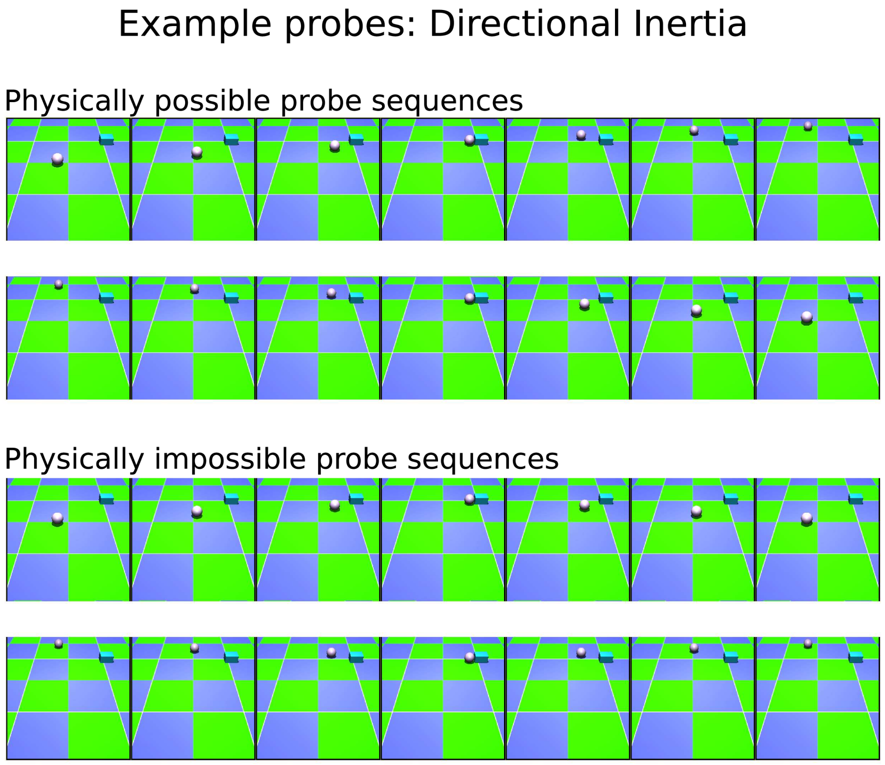

Supplementary Figure 4: Example probes adapted from (7) to assess the physical concept of directional inertia: colliding objects undergo changes to the direction of their velocity in accordance with the angle of incidence. Each row corresponds to one temporally down-sampled video in a probe tuple. Actual videos consist of a total of 15 frames. The top two rows are physically possible probes and the bottom two rows are physically impossible probes. **Physically Possible Probes**: In the first physically possible probe (first row), a ball rolls towards a block, ricochets off the block (which reverses its horizontal velocity) and maintains its movement towards the rear of the scene. The second possible probe (second row), reverses this trajectory: it starts at the rear of the scene, ricochets horizontally off the block, and continues to roll towards the front of the scene. **Physically Impossible Probes**: These probes are formed by splicing parts of the physically possible probes into impossible events. In the first impossible probe (third row), the ball rolls from the front of the scene towards the rear. When it collides with the block, instead of continuing towards the back of the scene, it reverses its direction back to the front of the scene. Similarly, in the second impossible probe (fourth row), when the ball collides with the block, it unexpectedly rolls back in the direction it came from.

Quantitative Analysis

Below we report the exact values depicted in various figures in the main manuscript, along with statistical hypothesis tests to support our claims in the main manuscript.

Results for Figure 5 in Main Text

These results analyze the benefit of object representations and computations (PLATO) over "flat" baselines (FLAT: Equal Parameters (FEP), FLAT: Equal Capacity (FEC)).

Average Accuracy

Physical Concept	Model	Mean	Standard Deviation	t(4)	p
Continuity	PLATO	0.891	0.028	27.725	5.0e-6
	Flat: Equal Parameters	0.548	0.039	2.479	0.034
	Flat: Equal Capacity	0.705	0.145	2.829	0.024
Directional Inertia	PLATO	0.727	0.017	26.947	5.6e-6
	Flat: Equal Parameters	0.452	0.026	-3.661	0.989
	Flat: Equal Capacity	0.694	0.204	1.903	0.065
Object Persistence	PLATO	0.678	0.043	8.248	5.9e-4
	Flat: Equal Parameters	0.502	0.01	0.370	0.365
	Flat: Equal Capacity	0.509	0.016	1.091	0.168
Solidity	PLATO	0.719	0.064	6.810	0.001
	Flat: Equal Parameters	0.365	0.213	-1.268	0.863
	Flat: Equal Capacity	0.503	0.036	0.174	0.435
Unchangeableness	PLATO	0.656	0.021	14.701	6.2e-5
	Flat: Equal Parameters	0.538	0.042	1.810	0.072
	Flat: Equal Capacity	0.493	0.115	-0.129	0.548

Supplementary Figure 5: Results of a one-sample, one-sided t-test against the null hypothesis that average accuracy across all five training seeds is not greater than chance accuracy (0.5).

**Mean Relative Surprise**					
		Mean	Standard Deviation	t(4)	p
Physical Concept	Model				
Continuity	PLATO	0.044	0.006	15.898	4.6e-5
	Flat: Equal Parameters	4.3e-4	4.4e-4	1.950	0.061
	Flat: Equal Capacity	0.009	0.008	2.378	0.038
Directional Inertia	PLATO	0.017	7.0e-4	47.790	5.7e-7
	Flat: Equal Parameters	2.1e-4	0.001	0.287	0.394
	Flat: Equal Capacity	0.012	0.01	2.433	0.036
Object Persistence	PLATO	0.034	0.008	8.709	4.8e-4
	Flat: Equal Parameters	6.5e-4	5.7e-4	2.274	0.043
	Flat: Equal Capacity	-3.9e-5	6.4e-5	-1.218	0.855
Solidity	PLATO	0.009	0.003	6.345	0.002
	Flat: Equal Parameters	-5.0e-4	6.2e-4	-1.611	0.909
	Flat: Equal Capacity	9.3e-5	2.0e-4	0.916	0.206
Unchangeableness	PLATO	0.007	2.2e-4	60.571	2.2e-7
	Flat: Equal Parameters	2.3e-4	1.9e-4	2.412	0.037
	Flat: Equal Capacity	1.4e-4	1.3e-4	2.154	0.049

Supplementary Figure 6: Results of a one-sample, one-sided t-test against the null hypothesis that the mean relative surprise across five training seeds is greater than 0.

Results for Figure 6 in Main Text

These results analyze the impact of training set size (i.e. amount of visual experience) on PLATO's performance.

**Grand Mean: Average Accuracy**				
	Mean	Standard Deviation	t(2)	p
Training Set Size				
0K	0.504	0.007	0.849	0.243
10K	0.644	0.101	2.020	0.09
50K	0.752	0.015	23.266	9.2e-4
100K	0.753	0.023	15.233	0.002
200K	0.756	0.022	16.564	0.002
300K	0.724	0.007	44.567	2.5e-4

Supplementary Figure 7: Here we present quantitative analysis of the grand mean across all physical concepts in our training data. These are the results of a one-sample, one-sided t-test against the null hypothesis that the average accuracy across is greater than 0. We perform this analysis using three different training seeds.

**Grand Mean: Mean Relative Surprise**				
	Mean	Standard Deviation	t(2)	p
Training Set Size				
0K	0.000	0.000	0.505	0.332
10K	0.012	0.008	2.011	0.091
50K	0.020	0.003	9.246	0.006
100K	0.021	0.003	10.974	0.004
200K	0.023	0.003	12.630	0.003
300K	0.022	0.001	45.969	2.4e-4

Supplementary Figure 8: Here we present analysis of the grand mean across all physical concepts in our training data. These are the results of a one-sample, one-sided t-test against the null hypothesis that the mean relative surprise across is greater than 0. We perform this analysis using three different training seeds.

Results for Figure 7 in Main Text

These results analyze PLATO's performance on three adapted ADEPT datasets.

**PLATO: Average Accuracy on ADEPT Data**				
	Mean	Standard Deviation	t(4)	p
Physical Concept				
Block (ADEPT)	0.765	0.049	10.917	2.0e-4
Overturn Long (ADEPT)	0.965	0.037	25.311	7.2e-6
Overturn Short (ADEPT)	0.79	0.163	3.566	0.012

Supplementary Figure 9: Results of a one-sample, one-sided t-test against the null hypothesis that average accuracy across all five training seeds is not greater than chance accuracy (0.5).

**PLATO: Mean Relative Surprise on ADEPT Data**				
	Mean	Standard Deviation	t(4)	p
Physical Concept				
Block (ADEPT)	0.007	0.002	7.514	8.4e-4
Overturn Long (ADEPT)	0.069	0.011	12.671	1.1e-4
Overturn Short (ADEPT)	0.022	0.016	2.822	0.024

Supplementary Figure 10: Results of a one-sample, one-sided t-test against the null hypothesis that the mean relative surprise across five training seeds is greater than 0.

Computing Interactions

The *InteractionNetwork* used in the *InteractionLSTM* computes two types of interactions. It computes pairwise interactions from the *ComponentLSTM*'s cell state ($cell_{1:K}^{t-1}$) to the *ComponentLSTM*'s cell state via an MLP with weights ρ. It also computes pairwise interactions using an MLP with weight λ from the *ComponentLSTM*'s cell state to a non-linear projection of the input object buffer codes $z_{1:K}^{1:t}$). The two interaction types are summed for each ordered pair and subsequently aggregated to form a single "interaction vector" for each slot in the ComponentLSTM. For maximum clarity, we include pseudocode below:

```
for k = 1 to K:
  for j = 1 to K:
    // Compute interactions from LSTM slots
    // to LSTM slots.
    cell2cell[k,j] = MLP_ρ(concat([cell_k^{t-1}, cell_j^{t-1}])

    // Compute interactions from LSTM slots
    // to projection of object inputs using
    // Exponential Linear Unit (ELU) non-linearity.
    cell2in[k,j] = MLP_λ(concat([cell_k^{t-1}, ELU(Linear(z_j^{1:t}])))

    //Combine interaction types.
    interaction[k,j] = cell2cell[k,j] + cell2in[k,j]

  //Aggregate all interactions for each object.
  interaction[k] = concat(sum(int[k,:]), max(int[k,:]))
```

Hyperparameter Selection

We chose PLATO's hyperparameters by evaluating our model's predictive performance on a held-out portion of our 'Freeform' dataset. Although the combinatorial space of hyperparameters did not allow systematically isolating each hyperparameter, below we report results for hyperparameters that seemed most impactful throughout the development of PLATO.

Does the object buffer need the full history of each object?

The object buffer, $z_{1:K}^{1:t}$, is used as an input to the *InteractionLSTM* at each timestep t of a video sequence. It is an input to both the *InteractionNetwork* and again to the *ComponentLSTM*. It represents the history of each object seen so far. However, given that LSTMs have hidden and cell states which can retain information over time, it is unclear whether or not we need the full history at each timestep. To investigate this issue, we performed a hyperparameter search over the number of input frames at each timestep. On one end, the object buffer

can contain just the current frame: $z^t_{1:K}$. On the other end, the object buffer can contain all previously seen frames $z^{1:t}$. In between, we investigated feeding in up to 8 of the most recent frames. The training curves below show that pixel-level predictions improved as we increased the number of frames in the object buffer. This was also inline with our informal assessment: including the full object history gave more plausible next-step predictions. Although we do not make claims about the biological plausibility of our implementation, using the "full history" in our dataset corresponds to a memory of approximately three seconds which is within the capabilities of humans.

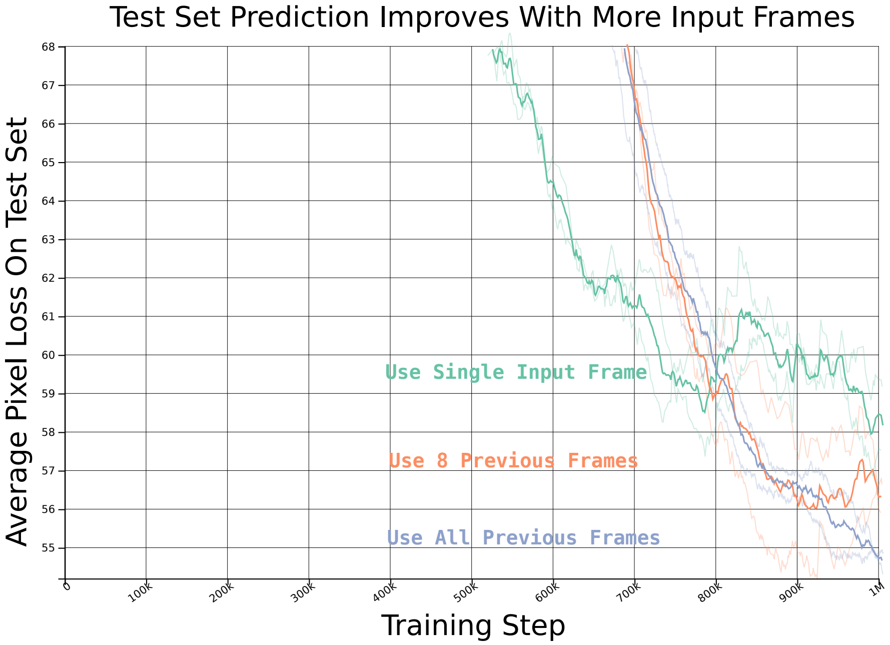

Supplementary Figure 11: The network makes better predictions when fed more of the input history. The y-axis shows the pixel-level prediction error on a held-out portion of our 'Freeform' training data averaged per-frame. We tried feeding in just the current input frame (green), all of the previously seen input frames (purple), or a maximum of eight input frames (orange). Faint lines represent individual seeds, dark lines represent average over seeds.

Does the dynamics predictor need recurrence?

Given that we decided to use the full object history in PLATO's object buffer, we investigated whether we needed recurrence at all in the dynamics predictor. Recall that the dynamics predictor uses an *InterationLSTM*: a combination of an InteractionNetwork and a ComponentLSTM. Since the full object history was present, arguably recurrent processing wasn't needed to retain information about occluded objects. We built a version of PLATO where we replaced the ComponentLSTM with a multi-layer perceptron (MLP). We call this version

the IntMLP. We found that this significantly reduced our model's predictive accuracy as seen in the curves below compared to using an LSTM.

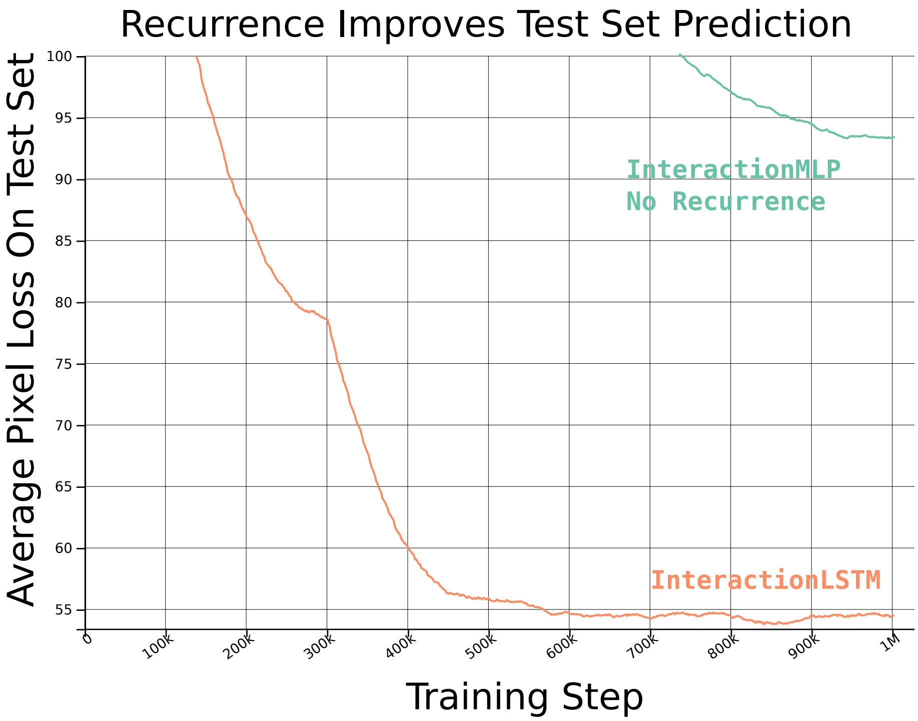

Supplementary Figure 12: The network makes better predictions when the dynamics predictor includes recurrence. The y-axis shows the pixel-level prediction error on a held-out portion of our 'Freeform' training data averaged per-frame. Despite the fact that the object buffer contains the full object history in these models (and therefore recurrence is not strictly needed), the model with recurrence (green) outperforms the model without recurrence (orange). Faint lines represent individual seeds, dark lines represent average over seeds.

Furthermore, we investigated the impact of recurrence on VoE effects for our test probes. Despite having access to the full history of frames in the object buffer, the non-recurrent model fared poorly on probes with long occlusions: *object persistence* and *'unchangeableness'*.

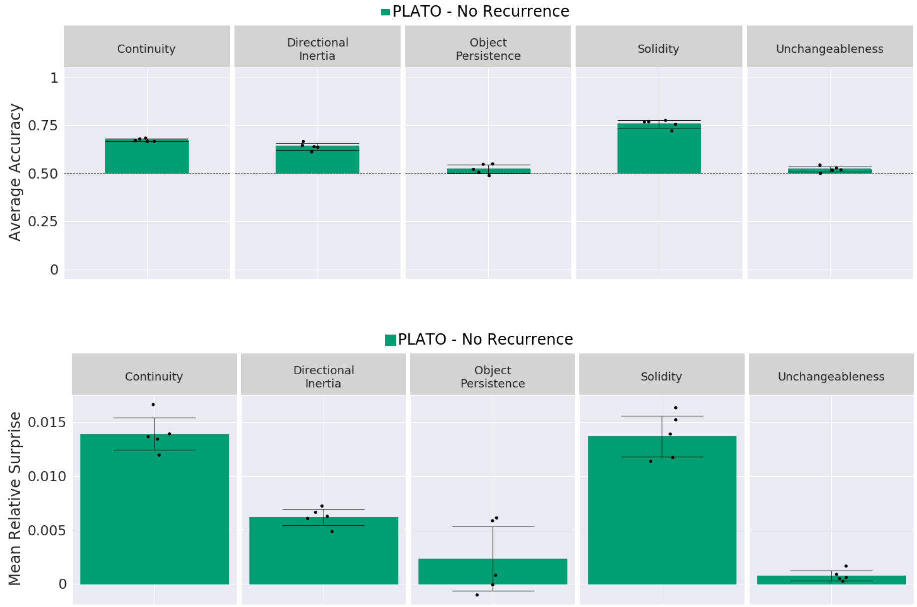

Supplementary Figure 13: Effect of Removing Recurrence From Dynamics Predictor in PLATO. Bars show the mean over five random training seeds and error bars depict standard error of the mean (SEM). Instead of using a slotted LSTM, the above results were generated by using a "slotted," 2056-unit, feedforward layer with shared weights. Even without recurrence this model does better than the flat baselines, but it is also evident that recurrence helps on probes with longer occlusion periods (*object persistence* and *'unchangeableness'*).

Does the LSTM in the InteractionLSTM need to be so large?

During development of the model, we explored how the number of hidden units in the LSTM impacted predictive performance. The pattern that emerged was clear: performance on the 'Freeform' test set improved as we increased the number of hidden units. Hardware limitations prevented us from increasing the number of hidden units beyond 2056 without introducing complex parameter partitioning schemes. However, we suspect the predictive performance to improve as we continued to increase the number of parameters.

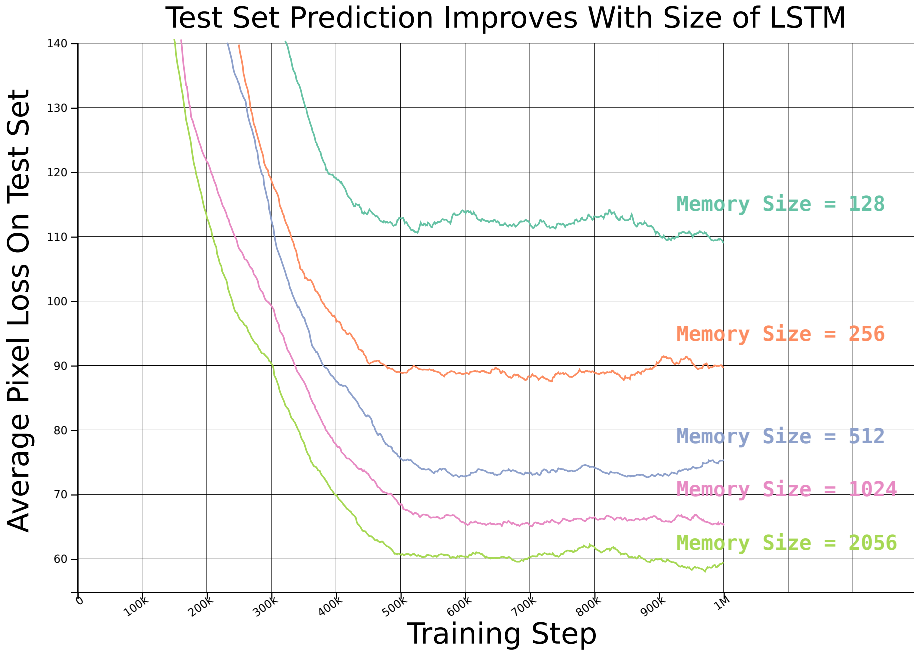

Supplementary Figure 14: Pixel-level prediction improves with size of LSTMs.

How does the projection size for the object buffer impact performance?

Along the development of PLATO, we hypothesized that learning an embedding space for the object buffer history could be beneficial. Instead of feeding $z^k_{1:t}$ directly to the InteractionLSTM, we learned a non-linear projection into a higher-dimensional space using the Exponential Linear Unit activation function. As shown below, increasing the size of the embedding space vastly improved pixel-level prediction. The worst model in pink has no input projection at all. As we increase the size of the embedding space performance improves on test set prediction. Note: for this comparison we used only 512 hidden units in the LSTM as opposed to the full size of 2056 hidden units.

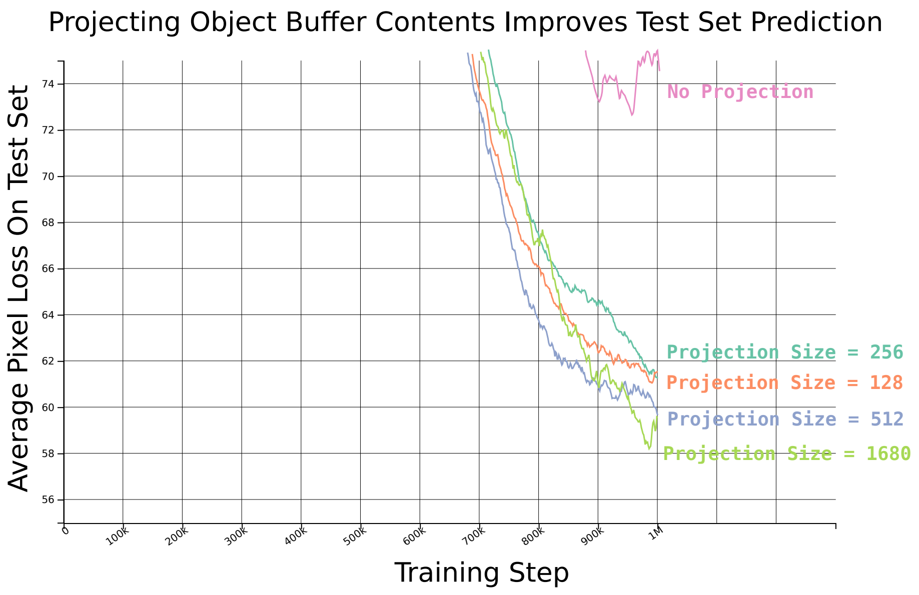

Supplementary Figure 15: Projecting the object buffer before sending to the InteractionLSTM improves predictions.

Additional VoE Analyses

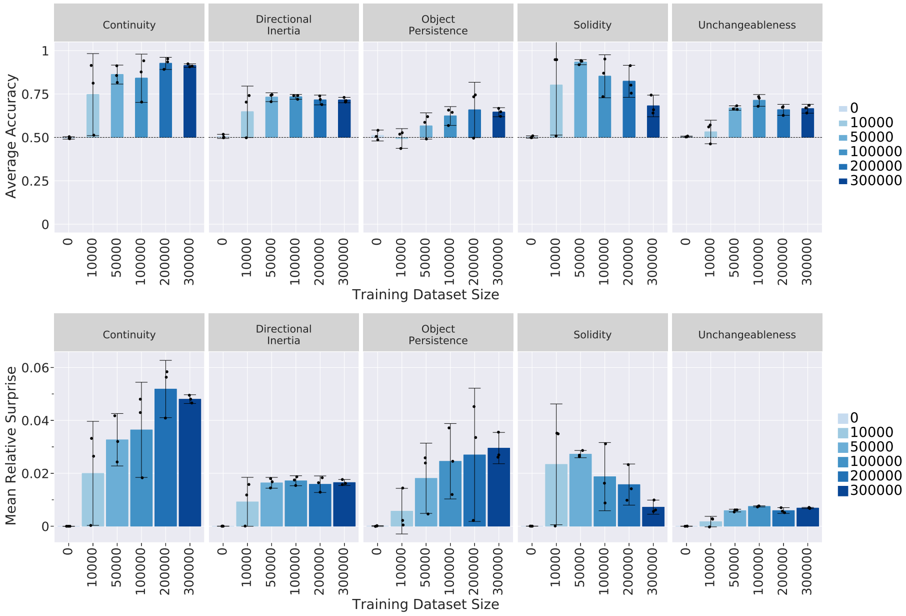

Supplementary Figure 16: Effect of Varying Training Set Size For Individual Physical Concepts. Bars show the mean over three random training seeds and error bars depict standard error of the mean (SEM).

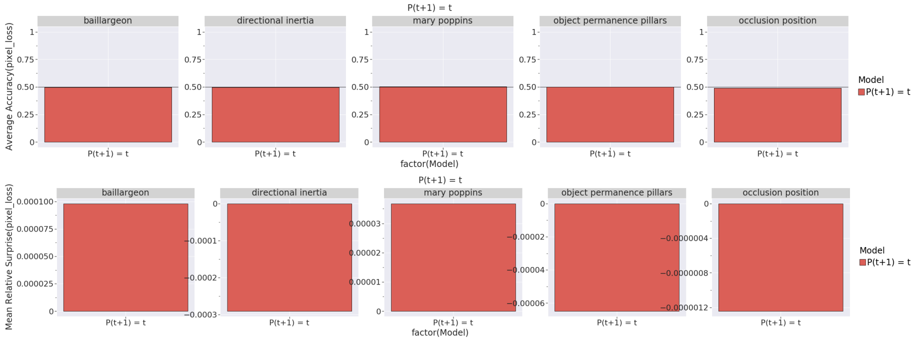

Supplementary Figure 17: Naive “predictor” baseline used to verify no biases in our probes. This is the result of replacing our dynamics predictor with a module that simply outputs the current input object codes as its prediction for the next timestep. There is no learning at all in this “predictor”, but it does use representations from the perceptual module. This figure serves as an empirical validation there are no systematic biases in our probes. Recall that the physically possible and physically impossible probes are controlled at the level of images (for any probe tuple, all the images contained in the possible probes appear exactly the same number of times in the impossible probes). However, we also control the probes at the level of *pairs* of images – every transition between two images that occurs in the possible probes also appears in the impossible probes. Thus, we provide this model that “predicts” the previous frame as its current input as a way of testing that the pairs of images are not systematically biased to make it easier or harder to see VoE effects from our probe dataset.

Representations Learned by Perception Module

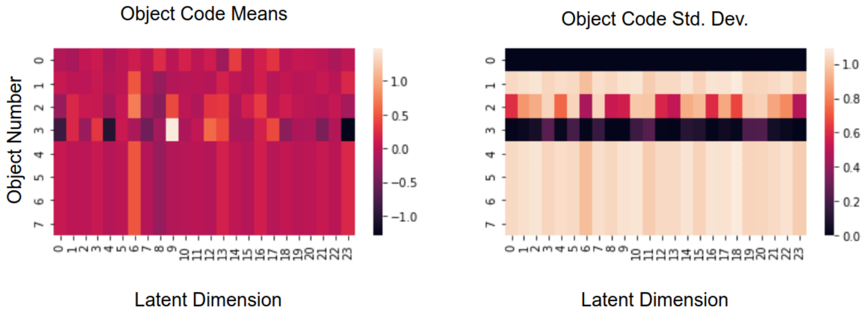

Supplementary Figure 18: Perceptual module posterior when there are "empty" object slots. **Left** Mean of posterior. **Right** Standard deviation of posterior distribution. Rows correspond to different object slots for a total of $K = 8$ slots. Columns correspond to different dimensions of the latent space. As mentioned in the main text, because of the high frequency of empty slots – most scenes do not have the maximum number of objects (eight) in them – the perceptual module assigns the unit Gaussian to these empty slots. This is evidenced in the above figure where slots 1, 4, 5, 6, 7 are empty and have the highest standard deviation across every single latent dimension. Slots with objects in them tend to have a much lower standard deviation and as such are properly predictable. As such, taking the latent loss is highly biased towards the unpredictable "empty" slots, which motivated our use of a pixel loss for evaluating our probes.

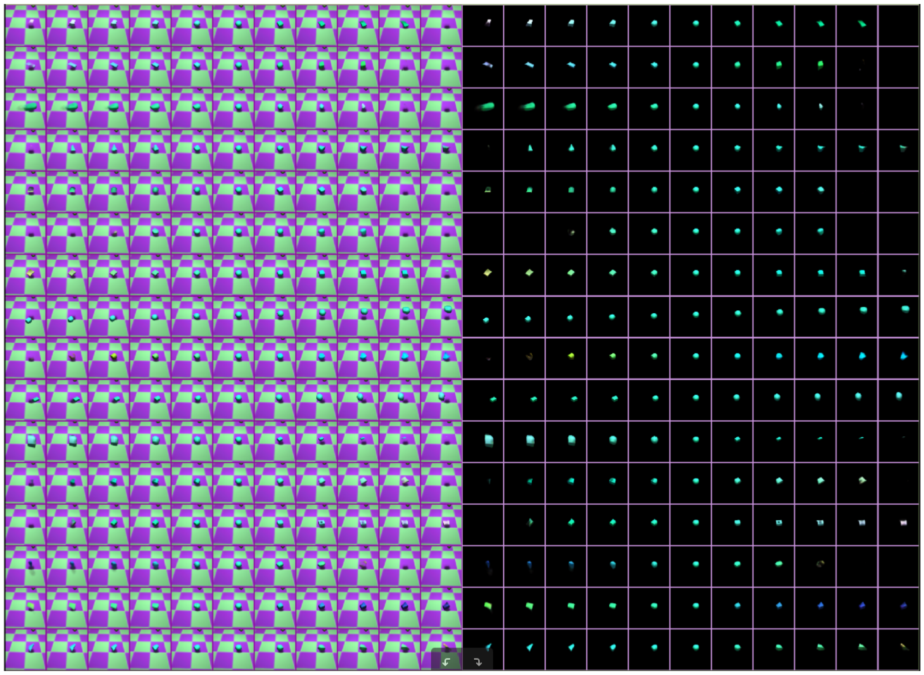

Supplementary Figure 19: Visualization of learned representational space for object codes. Generated via traversing the latent space learned in the perceptual module. A segmented image is encoded to form a set of $K = 8$, 16-dimensional posteriors and we get a latent code by sampling from that posterior. Then, we systematically perturb each dimension of each (non-empty) latent code. Each perturbed latent code is run through the perceptual module's decoder to yield an image. We visualize this image either as the entire scene (left half) or rendering only the perturbed object in isolation (right half). Each row corresponds to perturbing a different dimension of the latent code. The center column of each half depicts the unperturbed latent. Here we visualize the perturbation for just the single blue sphere in the image. Although the axes are not perfectly disentangled, we can see perturbations of various axes yield changes in high-level object properties such as shape (top row: ranging from a cube to a ramp), color (various rows), height off ground (eighth row).

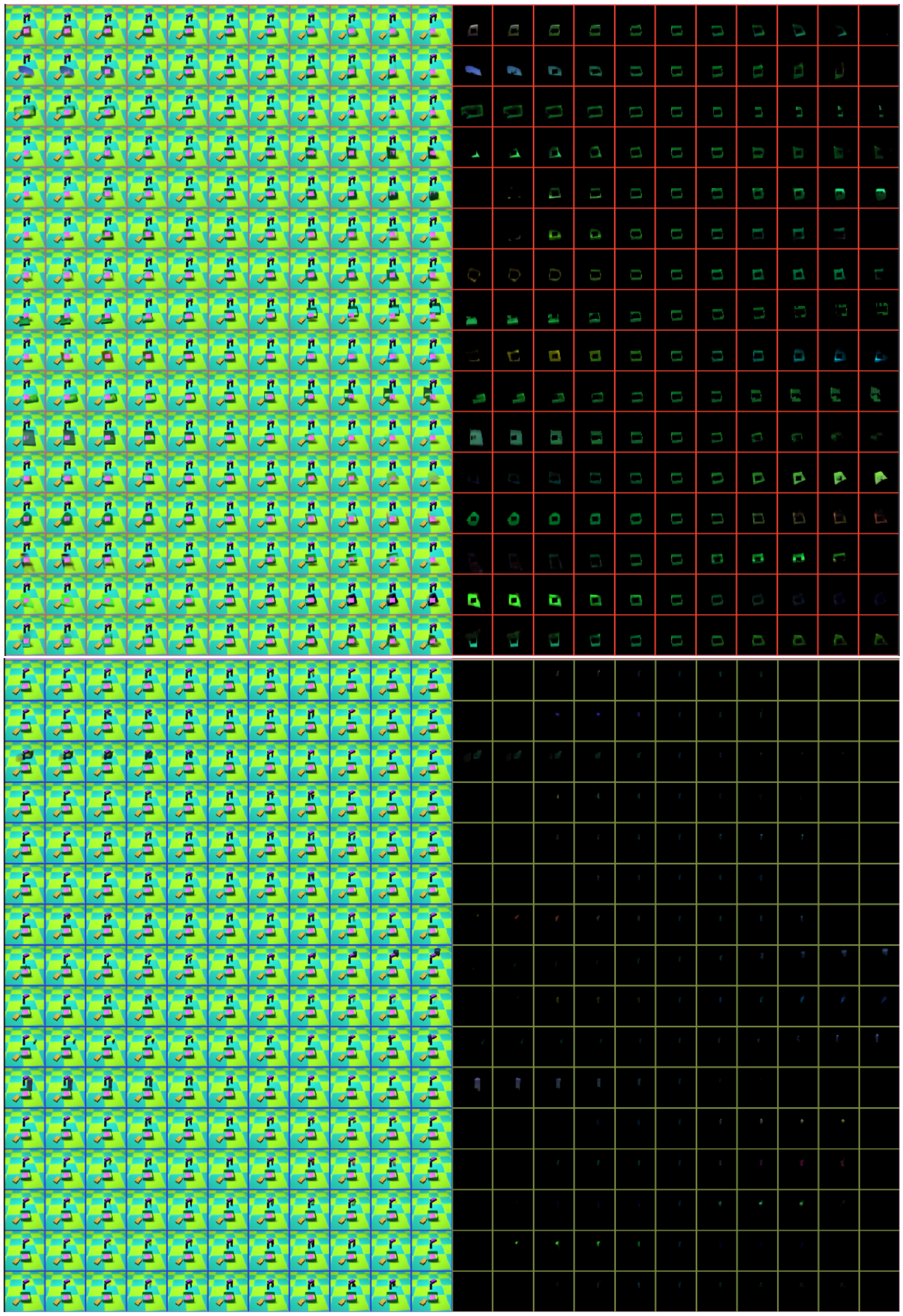

Supplementary Figure 20: Latent traversals for a busier scene, including traversals generated by the object code for the floor.

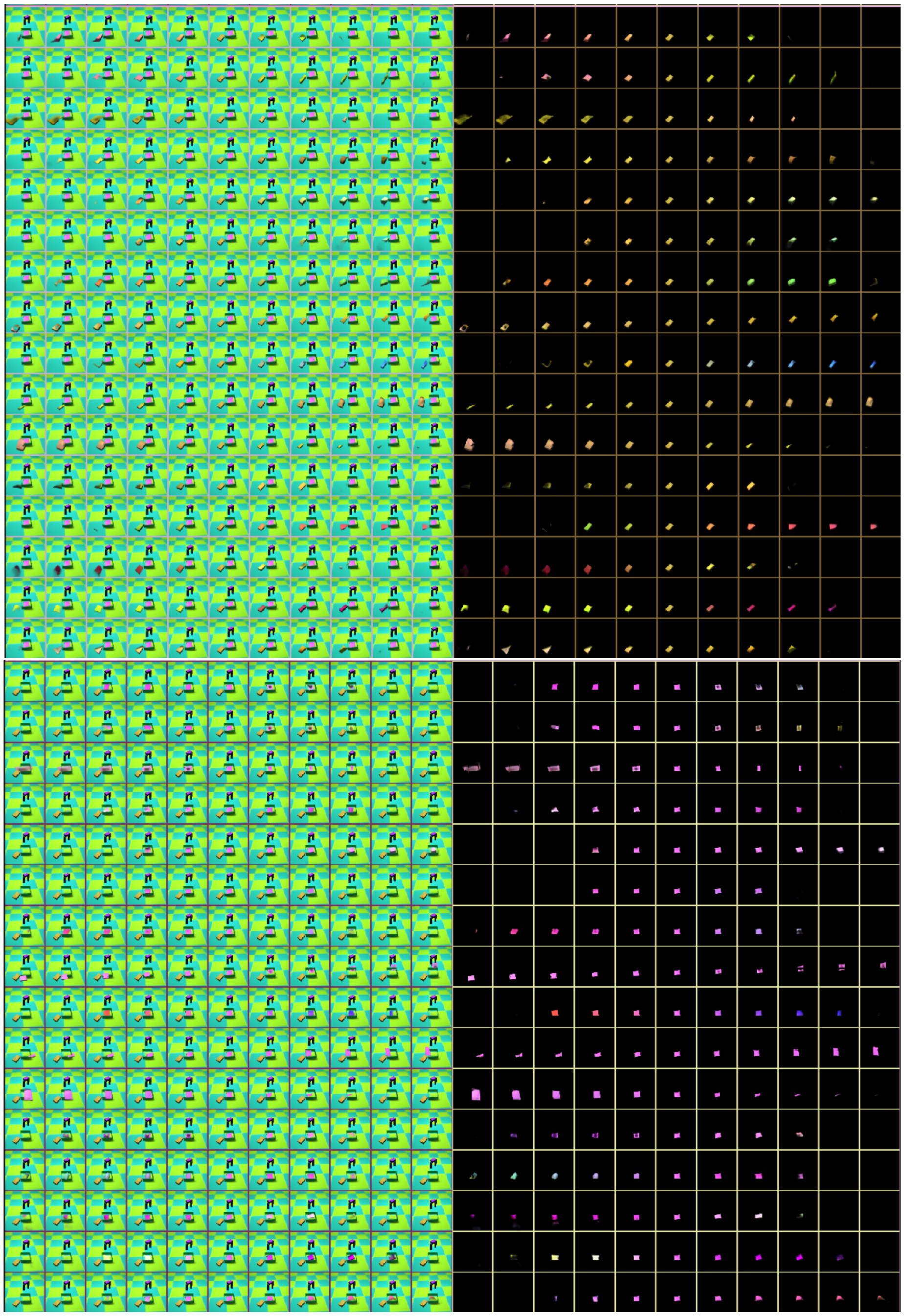

Supplementary Figure 21: Continuation of latent traversals above.

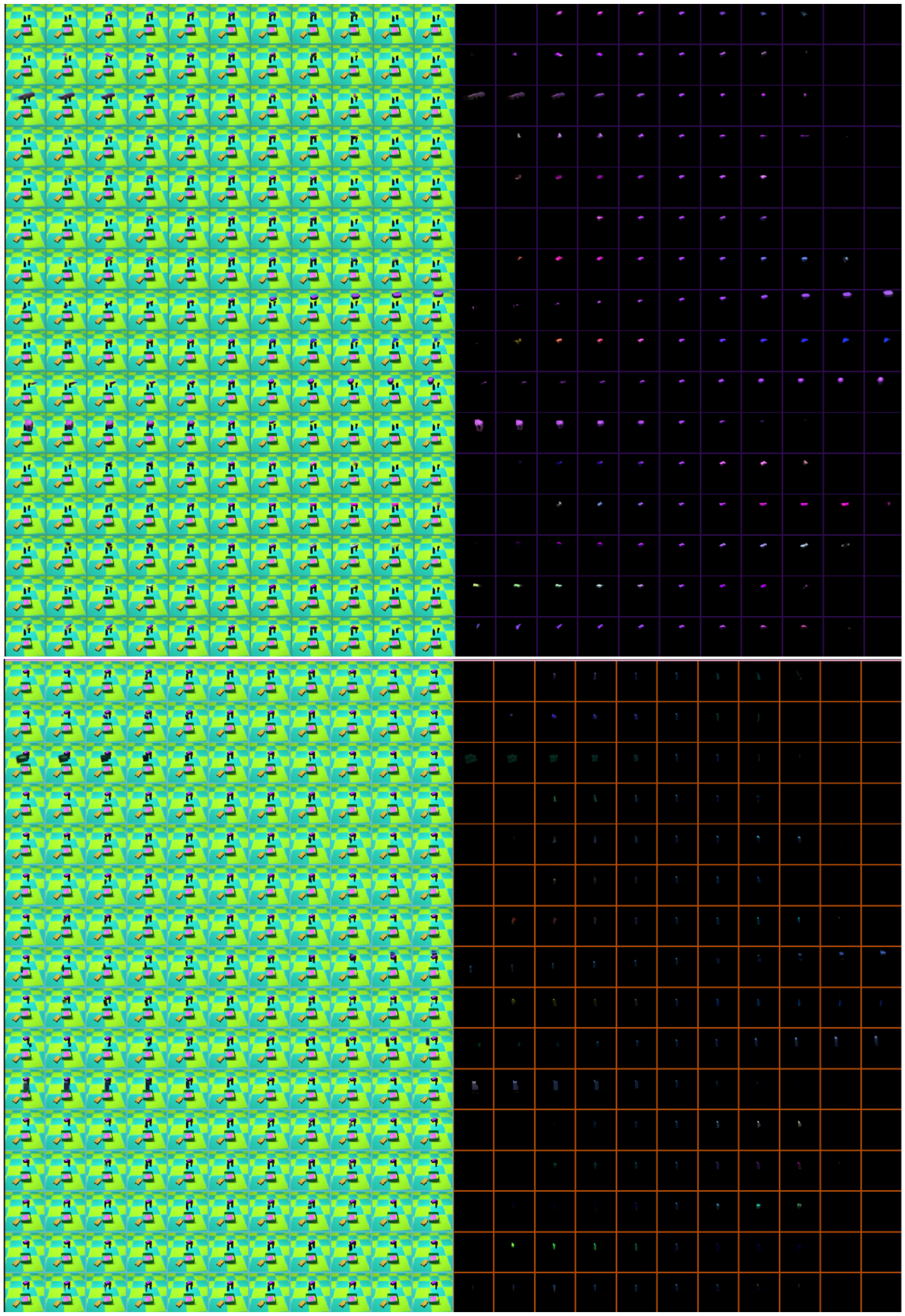

Supplementary Figure 22: Continuation of latent traversals above

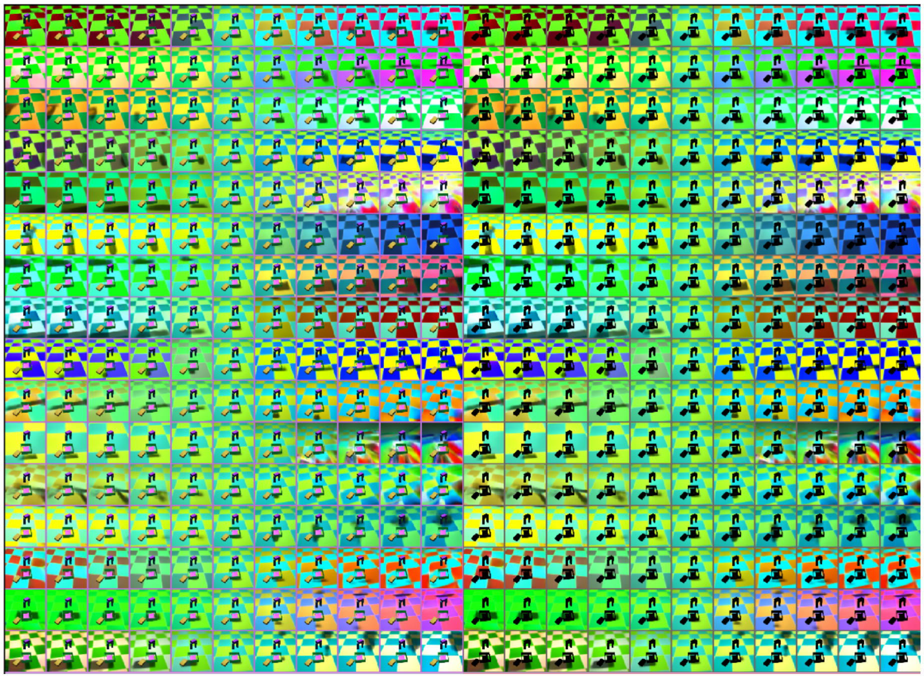

Supplementary Figure 23: Continuation of latent traversals above - traversal for object code representing the ground object.

Model Predictions

In the Supplementary Materials, we have included visualizations of PLATO's predictions for the "Freeform" data under two conditions: next-step prediction and rollouts. In the next-step prediction condition, we generate videos showing PLATO's predictions given input frames from the dataset. In the rollouts condition, halfway through a video from the dataset we start feeding PLATO's predictions as the inputs. In our videos, we visualize both the targets (top image row) and predictions (bottom image row) as entire composite images (columns 1 and 2) and also as individual object slots (columns 3 to 8), where the floor counts as an "object". An all black cell indicates there was no visible object in that slot at that particular frame.

Careful inspection of the supplementary videos shows that PLATO makes reasonable next-step predictions and captures qualitatively important event features like predicting changes in trajectories following a collision or emerging from occlusion. On the other hand, PLATO's rollouts are frequently unstable. This is inline with our expectations: PLATO was trained for next-step prediction. In our experience, training models that produce consistent and stable rollouts requires its own optimization and regularization methods – especially when those trajectories are over a learned representational space like the VAE latents.

It is worth noting that the literature provides no information about what the children studied in developmental experiments are able to 'picture in their mind's eye'. However, the results we report show that robust VoE effects are possible without an accompanying ability to generate very precise multi-step predictions. Of course, there is also a lot of other work in deep learning showing that deep learning systems can nonetheless learn to make such predictions under the right circumstances (Sanchez-Gonzalez, Alvaro, et al. "Learning to simulate complex physics with graph networks." International Conference on Machine Learning. PMLR, 2020.)

References

[1] Baillargeon, R. Object permanence in 31/2-and 41/2-month-old infants. *Developmental psychology* **23**, 655 (1987).

[2] Baillargeon, R. Innate ideas revisited: For a principle of persistence in infants' physical reasoning. *Perspectives on Psychological Science* **3**, 2–13 (2008).

[3] Hespos, S. J. & Baillargeon, R. Infants' knowledge about occlusion and containment events: A surprising discrepancy. *Psychological Science* **12**, 141–147 (2001).

[4] Káldy, Z. & Sigala, N. The neural mechanisms of object working memory: what is where in the infant brain? *Neuroscience & Biobehavioral Reviews* **28**, 113–121 (2004).

[5] Baillargeon, R. & Carey, S. Core cognition and beyond: The acquisition of physical and numerical knowledge. *Early childhood development and later outcome* 33–65 (2012).

[6] Spelke, E. S., Breinlinger, K., Macomber, J. & Jacobson, K. Origins of knowledge. *Psychological review* **99**, 605 (1992).

[7] Spelke, E. S., Katz, G., Purcell, S. E., Ehrlich, S. M. & Breinlinger, K. Early knowledge of object motion: Continuity and inertia. *Cognition* **51**, 131–176 (1994).
