# Supplementary material for: Intuitive physics learning in a deep-learning model inspired by developmental psychology
Source: Nat Hum Behav. 2022 Jul 11;6(9):1257–67. doi: 10.1038/s41562-022-01394-8 (PMC9489531; doi:10.1038/s41562-022-01394-8)
Supplement: Supplementary file 2 — Reporting Summary [file 41562_2022_1394_MOESM2_ESM.pdf]

nature research

Corresponding author(s): LUIS S. PILOTO

Last updated by author(s): May 12, 2022

Reporting Summary

Nature Research wishes to improve the reproducibility of the work that we publish. This form provides structure for consistency and transparency in reporting. For further information on Nature Research policies, see our Editorial Policies and the Editorial Policy Checklist.

Statistics

For all statistical analyses, confirm that the following items are present in the figure legend, table legend, main text, or Methods section.

n/a	Confirmed	
☐	☒	The exact sample size (*n*) for each experimental group/condition, given as a discrete number and unit of measurement
☐	☒	A statement on whether measurements were taken from distinct samples or whether the same sample was measured repeatedly
☒	☐	The statistical test(s) used AND whether they are one- or two-sided<br>*Only common tests should be described solely by name; describe more complex techniques in the Methods section.*
☒	☐	A description of all covariates tested
☒	☐	A description of any assumptions or corrections, such as tests of normality and adjustment for multiple comparisons
☐	☒	A full description of the statistical parameters including central tendency (e.g. means) or other basic estimates (e.g. regression coefficient) AND variation (e.g. standard deviation) or associated estimates of uncertainty (e.g. confidence intervals)
☒	☐	For null hypothesis testing, the test statistic (e.g. *F*, *t*, *r*) with confidence intervals, effect sizes, degrees of freedom and *P* value noted<br>*Give P values as exact values whenever suitable.*
☒	☐	For Bayesian analysis, information on the choice of priors and Markov chain Monte Carlo settings
☒	☐	For hierarchical and complex designs, identification of the appropriate level for tests and full reporting of outcomes
☐	☒	Estimates of effect sizes (e.g. Cohen's *d*, Pearson's *r*), indicating how they were calculated

Our web collection on statistics for biologists contains articles on many of the points above.

Software and code

Policy information about availability of computer code

Data collection	We did not collect data, but a core aspect of this work is the synthetic dataset we generated. As described in the text, we used a combination of custom "Python" (v 3.7.6) programming scripts and the Mujoco (v2.0) physics engine to generate our dataset. The custom scripts were written to populate the Mujoco physics engine with various conditions and run these simulations over an internal distributed computing cluster. We make the generated dataset fully available.
Data analysis	We provide a Python Colab notebook suitable for loading our synthetic dataset into TensorFlow (v1). We used Tensorflow (v1) to build our deep learning model and custom Python scripts for distributed training over our internal computing infrastructure. We do not provide a specific implementation of our deep neural network and baselines, as there is no publicly-available, standard way to train/run our relatively large model or the two-phase training approach where we train on representations first and then use those representations for learning dynamics. However, we very thoroughly describe how we build our model from existing, well-known components. Please reach out to the corresponding author for any questions on implementation details.

For manuscripts utilizing custom algorithms or software that are central to the research but not yet described in published literature, software must be made available to editors and reviewers. We strongly encourage code deposition in a community repository (e.g. GitHub). See the Nature Research guidelines for submitting code & software for further information.

Data

Policy information about availability of data

All manuscripts must include a data availability statement. This statement should provide the following information, where applicable:

- Accession codes, unique identifiers, or web links for publicly available datasets
- A list of figures that have associated raw data
- A description of any restrictions on data availability

We make available (https://github.com/deepmind/physical_concepts/) our full synthetic dataset consisting of the training data and the various types of test data in our Physical Concepts dataset. Additionally, the ADEPT dataset is available at: http://physadept.csail.mit.edu/

Field-specific reporting

Please select the one below that is the best fit for your research. If you are not sure, read the appropriate sections before making your selection.

☒ Life sciences ☐ Behavioural & social sciences ☐ Ecological, evolutionary & environmental sciences

For a reference copy of the document with all sections, see nature.com/documents/nr-reporting-summary-flat.pdf

Life sciences study design

All studies must disclose on these points even when the disclosure is negative.

Sample size	Because our probe data was procedurally generated and we were evaluating models (not humans), we could easily have large sample sizes. We used a sample size of 5,000 probes per physical concept, each consisting of 4 videos. This is similar in magnitude to previous iteration of this work (https://arxiv.org/pdf/1804.01128.pdf) which used 8,000 probes consisting of 2 videos.
Data exclusions	No data was excluded: we built our test dataset and reported our model's behavior on the entirety of the test dataset.
Replication	We trained our model using multiple random seeds (either 5 or 3) and reported our results over the random seeds with corresponding error bars in the manuscript. We did not attempt to replicate these results outside of the reported results.
Randomization	We used random seeds (either 5 or 3) for weight initialization to evaluate the robustness of our results to different optimization runs.
Blinding	Because our models were evaluated programmatically, there was no potential for blinding.

Reporting for specific materials, systems and methods

We require information from authors about some types of materials, experimental systems and methods used in many studies. Here, indicate whether each material, system or method listed is relevant to your study. If you are not sure if a list item applies to your research, read the appropriate section before selecting a response.

Materials & experimental systems

n/a	Involved in the study	
☒	☐	Antibodies
☒	☐	Eukaryotic cell lines
☒	☐	Palaeontology and archaeology
☒	☐	Animals and other organisms
☒	☐	Human research participants
☒	☐	Clinical data
☒	☐	Dual use research of concern

Methods

n/a	Involved in the study	
☒	☐	ChIP-seq
☒	☐	Flow cytometry
☒	☐	MRI-based neuroimaging
